# Conditional knockdown of *OsMLH1* to improve plant prime editing systems without disturbing fertility in rice

**DOI:** 10.1186/s13059-024-03282-y

**Published:** 2024-05-21

**Authors:** Xiaoshuang Liu, Dongfang Gu, Yiru Zhang, Yingli Jiang, Zhi Xiao, Rongfang Xu, Ruiying Qin, Juan Li, Pengcheng Wei

**Affiliations:** 1 College of Agronomy, Anhui Agricultural University, Hefei, 230036 People’s Republic of China; 2 Rice Research Institute, Anhui Academy of Agricultural Sciences, Hefei, 230031 People’s Republic of China; 3 Research Centre for Biological Breeding Technology, Advance Academy, Anhui Agricultural University, Hefei, 230036 People’s Republic of China

**Keywords:** Prime editing, DNA mismatch repair, Rice, Fertility, Cre recombinase

## Abstract

**Background:**

High-efficiency prime editing (PE) is desirable for precise genome manipulation. The activity of mammalian PE systems can be largely improved by inhibiting DNA mismatch repair by coexpressing a dominant-negative variant of MLH1. However, this strategy has not been widely used for PE optimization in plants, possibly because of its less conspicuous effects and inconsistent performance at different sites.

**Results:**

We show that direct RNAi knockdown of *OsMLH1* in an ePE5c system increases the efficiency of our most recently updated PE tool by 1.30- to 2.11-fold in stably transformed rice cells, resulting in as many as 85.42% homozygous mutants in the T_0_ generation. The high specificity of ePE5c is revealed by whole-genome sequencing. To overcome the partial sterility induced by *OsMLH1* knockdown of ePE5c, a conditional excision system is introduced to remove the RNAi module by Cre-mediated site-specific recombination. Using a simple approach of enriching excision events, we generate 100% RNAi module-free plants in the T_0_ generation. The increase in efficiency due to *OsMLH1* knockdown is maintained in the excised plants, whose fertility is not impaired.

**Conclusions:**

This study provides a safe and reliable plant PE optimization strategy for improving editing efficiency without disturbing plant development via transient MMR inhibition with an excisable RNAi module of MLH1.

**Supplementary Information:**

The online version contains supplementary material available at 10.1186/s13059-024-03282-y.

## Background

Over the past decade, the adoption of gene editing technology from prokaryotic CRISPR–Cas systems has greatly changed patterns of plant research. Engineered CRISPR systems have rapidly produced mutants, primarily those obtained by knocking out desired genes in numerous plant species. Because many valuable agricultural traits are associated with specific single-nucleotide polymorphisms (SNPs) or short variations, precise genome editing technologies for effectively manipulating nucleotide substitutions, deletions, and insertions are greatly needed [1]. Homology-directed repair (HDR) via CRISPR-induced DNA double-strand breaks (DSBs) enables precise editing of the genome by introducing defined changes in an exogenous donor template. However, the efficiency of precise plant genome editing through HDR is limited due to the extremely low intrinsic activity and delivery barriers associated with donor molecules [1]. To address this problem, several approaches, such as employing high amounts of donors provided by geminiviral replicon systems or transcript template systems to secure recombination events in rice [2, 3], sequentially delivering single guide RNAs (sgRNAs) and donors in germline-specific Cas9-expressing *Arabidopsis* lines [4], enriching donor-released events via the inducible Cas9 system in maize [5], and bombarding chemically modified donor DNA using tandem repeat HDR technology [6], have been exploited. Although these strategies have increased editing efficiency, methods relying on HDR are still plagued by intricated procedures and exhibit highly variable performance at different genomic loci [7].

Recently developed prime editing (PE) technology has revolutionized precise genome engineering by bypassing the need for HDR machinery [8]. A prime-editing guide RNA (pegRNA) directs a prime editor to nick the non-target strand (NTS) with a Cas9 H840A nickase in the fusion. The exposed 3′ flap hybridizes with the primer binding site (PBS) sequence of the pegRNA, which allows the reverse transcriptase (RT) of the editor to synthesize new single-strand DNA following the customized RT template (RTT). The 3′ flap extension displaces the adjacent genomic sequence through endonuclease excision on the unedited 5′ flap sequence. The heteroduplex intermediate of the 3′ flap strand and the target strand (TS) is resolved by a mismatch repair (MMR) system to install edits [9, 10]. However, the nicked edited strand is prone to excise by MMR and correct following the unedited TS. In mammalian cells, CRISPRi screening of DNA repair-related genes revealed that several MMR components, such as MLH1, MSH2, MSH3, MSH6, and PMS2, are involved in the regulation of PE efficiency [9, 10]. To manipulate MMR activity during editing, a dominant-negative MLH1 (MLH1dn) protein was coexpressed with PE2/PE3 systems. The average editing efficiency of the resulting PE4/PE5 systems increased 2.8- to 7.7-fold in a variety of cell types [9].

PE has been exploited to obtain desired mutations in monocots and dicots [11]. Although plant prime editors have been vastly optimized for installing base substitutions and small insertions and deletions (indels) [12], their applications remain restricted due to the relatively low efficiency in many scenarios. The strategy of MMR suppression has also been attempted in plant PE engineering. For instance, compared with ePE3max, ePE5max expressing a dominant-negative variant of rice MLH1 (*OsMLH1dn*) or maize MLH1dn (*ZmMLH1dn*) increased the proportion of homozygous mutations in stable transgenic lines [13, 14]. However, ePE5max using OsMLH1dn exhibited comparable but not significantly enhanced efficiency compared with ePE3max in protoplasts, and the same finding was obtained for the overall mutation efficiency in transgenic rice [13]. Similarly, our former study revealed that the fusion of *human MLH1dn* (*hMLH1dn*) in the pPE2max system did not outperform the original version in calli [15]. These findings suggest that the suppression of MMR activity via the use of dominant-negative MLH1 variants may be less effective in improving the activity of prime editors in plants than in mammals. In this study, we showed that direct knockdown of *OsMLH1* robustly enhanced rice PE efficiency.

## Results

### ePE3 was not obviously enhanced by the coexpression of MLH1dn orthologs

**Fig. 1:**
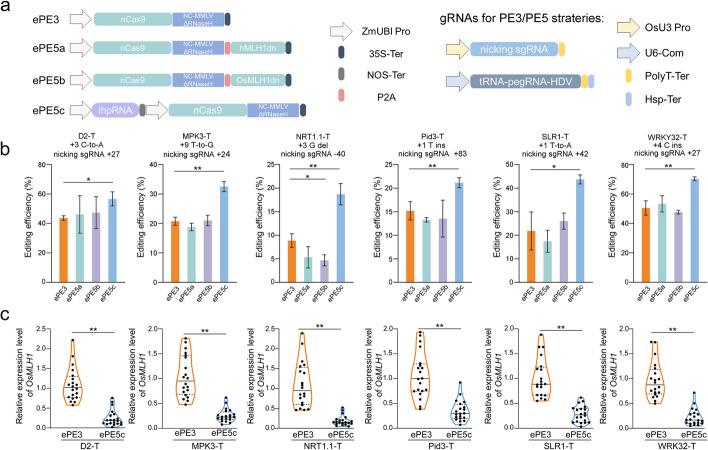
Prime editing of engineered PE systems in rice calli. **a** Diagram of the PE systems. Left, structure of the engineered editors. nCas9, H840A nickase of the SpCas9-R221K/N394K variant; NC-MMLVΔRNaseH, fusion of the viral NC protein and the RNase H domain-truncated version of the evolved Moloney murine leukemia virus RT mutant (D200N/L603W/T330P/T306K/W313F); ZmUBI pro, maize ubiquitin 1 promoter; 35S-Ter, CaMV 35S terminator; NOS-Ter, NOS terminator; P2A, porcine teschovirus-1 2A self-cleavage peptide. Right, cassette of PE RNAs. OsU3 Pro, rice U3 promoter for nicking sgRNA expression; U6-com, the pol II promoter enhanced the U6 promoter for pegRNA expression; PolyT-Ter, poly T terminator; Hsp-Ter, terminator of Hsp18.2. **b** Efficiency of precise editing in stably transformed calli. The mutation and the position relative to the cleavage point of nCas9, as well as the nicking sgRNA site, are shown. Approximately 200 newly emerged calli after 2 to 3 weeks of selection were collected for amplicon NGS. The efficiencies were calculated from the ratios of edited reads to total clean reads. Three independent transformants were evaluated from different *Agrobacterium* clones of a vector as biological replicates to generate mean values and standard deviations. One-way ANOVA was used to determine the significance of the differences between the PE systems. *P < 0.05; **, *P* < 0.01. **c** Relative expression levels of *OsMLH1* in calli of ePE3 and ePE5c. The abundance of the *OsMLH1* transcript in independent resistance events was determined by qRT–PCR analysis. The mean values of three technical replicates are shown as dots inside the violin plot (*n* = 20). The differences were assessed by paired *t* tests. **P < 0.01

To test whether dominant-negative variants of MLH1 can enhance the editing activity of our recently developed ePE2 system [16], hMLH1dn and OsMLH1dn were fused to the C-terminus of ePE2 with a porcine teschovirus-1 2A (P2A) self-cleaving peptide to construct ePE2-hMLH1dn and ePE2-OsMLH1dn, respectively (Fig. 1a). Then, pegRNAs were designed to induce precise mutations at six targets in the rice genome. The PE3 or PE5 strategy was applied to increase the efficiency of expressing additional sgRNAs to nick the non-edited strand. After *Agrobacterium*-mediated rice transformation, the efficiencies of ePE5a (with the ePE2-hMLH1dn editor) and ePE5b (with the ePE2-OsMLH1dn editor) were determined by next-generation sequencing (NGS) of the target-specific amplicons in transgenic calli. Similar to previous observations of MLH1dn-engineered pPE3max in protoplasts and pPE2max in calli [13, 15], no significant increase in editing efficiency was obtained with ePE5a or ePE5b compared with that obtained with ePE3 (*P* > 0.05; Fig. 1b). Moreover, the efficiency of ePE5b was 52.81% of that of ePE3 at the NRT1.1-T site (*P* < 0.05), suggesting that inappropriate expression of *OsMLH1dn* might hinder editing at certain targets.

### The PE efficiency was improved by RNAi suppression of OsMLH1

In the K562 cell line, the knockdown of *MLH1* or other MutSα–MutLα genes via CRISPRi technology increased PE efficiency [9]. Given that the CRISPRi system has not been well established in plants [17], an intron-containing hairpin RNA (ihpRNA) against *OsMLH1* (*OsMLH1*-ihpRNA) was constructed for RNA interference (RNAi) of the transcript (Additional file 1: Fig. S1). The expression cassette of *OsMLH1*-ihpRNA was integrated into the ePE3 system, yielding ePE5c (Fig. 1a). RNAi suppression was examined by quantitative reverse transcription–PCR (qRT–PCR) assays of independent ePE3 and ePE5c events in stably transformed calli. For the constructs with different pegRNAs, the average *OsMLH1-*knockdown efficiency of ePE5c ranged from 67.32 to 82.68% (Fig. 1c). Statistical analysis confirmed that the transcript abundance of *OsMLH1* was significantly lower in ePE5c cells than in ePE3 cells (*P* < 0.05).

Subsequently, the editing efficiencies in calli were determined using amplicon NGS. We found that ePE5c had the highest efficiency among the four PE systems at all tested targets, and the ratios of the precise edits obtained with ePE5c ranged from 18.71 to 70.32% (Fig. 1b). Unlike ePE5a and ePE5b, ePE5c exhibited improved efficiency compared with ePE3, with significant increases of 1.30-fold for a C-to-A mutation at the D2-T site, 1.56-fold for a T-to-G mutation at the MPK3-T site, 2.11-fold for a G deletion at the NRT1.1-T site, 1.39-fold for a T insertion at the Pid3-T site, 2.00-fold for a T-to-A mutation at the SLR1-T site, and 1.39-fold for a C insertion at the WRKY32-T site (*P* < 0.05). Averaging the data from the six targets, ePE5c precisely installed mutations in 40.52% of the reads, resulting in 1.51-, 1.57-, and 1.52-fold increases compared with the results obtained with ePE3, ePE5a, and ePE5b, respectively. Undesired indels are the majority of byproducts of PE3/PE5 systems. The NGS data showed that ePE5c and ePE3 yielded similar frequencies of byproduct indels at four targets (Additional file 1: Fig. S2), whereas the byproduct indel frequencies obtained with ePE5c at MPK3-T and SLR1-T were significantly lower than those obtained with ePE3 (*P* < 0.01). Therefore, the byproduct editing of ePE5c was not synchronously boosted with intended editing. Taken together, these results indicated that rice PE efficiency can be robustly enhanced via RNAi-mediated suppression of *OsMLH1*.

### Editing in T_0_ plants is enhanced by OsMLH1 suppression

**Fig. 2:**
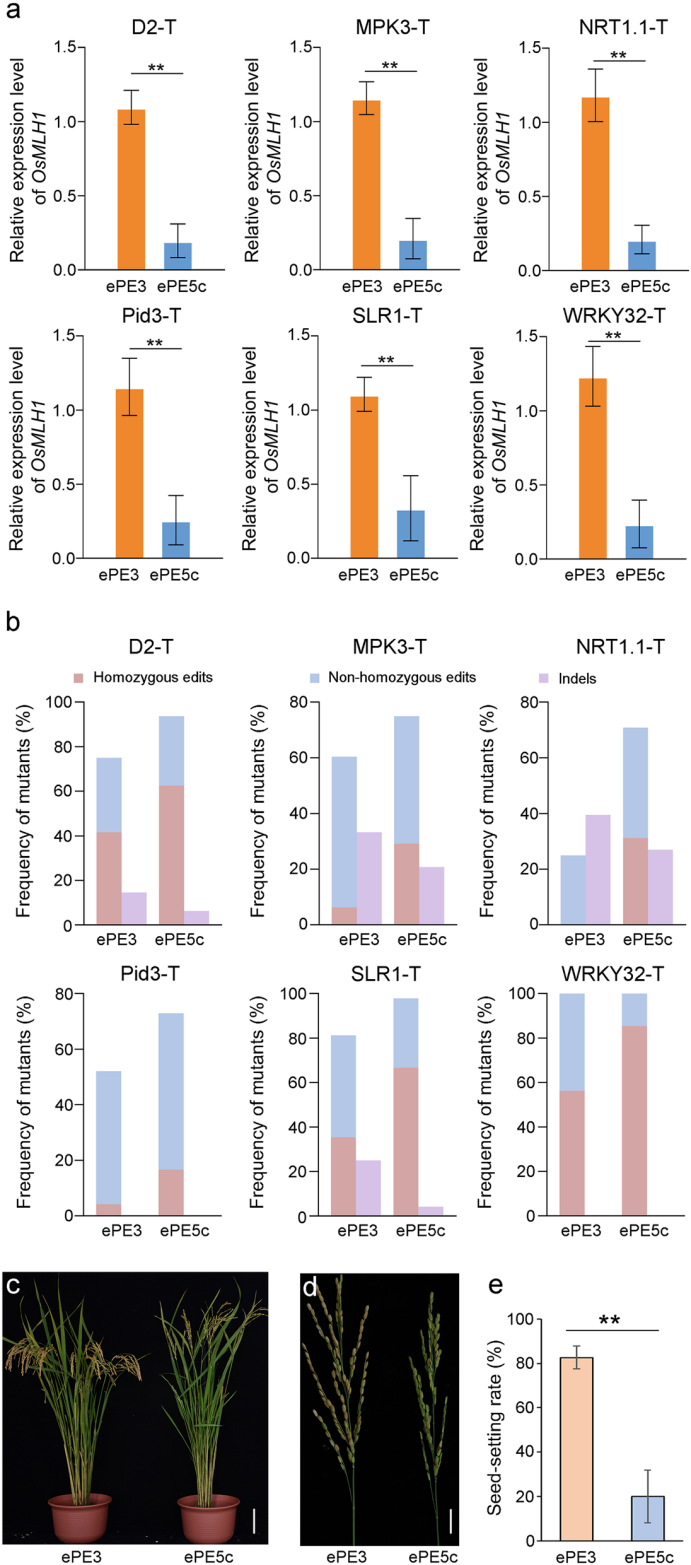
Prime editing of ePE3 and ePE5c in transgenic rice plants. **a** Relative expression levels of *OsMLH1* in T_0_ plants. The mean values and standard deviations of the qRT–PCR data were obtained from randomly selected independent lines with either the ePE3 (*n* = 4) or ePE5c (*n* = 12) vector. The differences were assessed by an independent *t* test. **P < 0.01. **b** Editing of the six targets in the T_0_ lines. For each vector, the target mutations were identified in 48 lines by the Hi-TOM assay. The ratio of plants harboring undesired indels to total plants is indicated in purple. The precise homozygous mutants are labeled in red, and the remaining plants harboring targeted mutations (non-homozygous edits, including heterozygous and chimeric edits) are labeled in blue. **c**, **d** Comparison of gross morphology and panicles of the ePE3 and ePE5c lines. Representative T_0_ lines harboring homozygous mutations in the WRKY32-T site (**c**) or their main panicle (**d**) are indicated at the yellow ripening stage. Scale bars = 10 cm (c) or 2 cm (d). **e** Statistical analysis of the seed-setting rates of the ePE3 and ePE5c lines. In an independent line, the seed-setting rate was averaged from at least three panicles. The T_0_ lines harboring homozygous mutations at the MPK3-T and WRKY32-T sites were randomly selected to generate means ± SDs (*n* = 24). The differences were assessed by paired *t* tests. **P < 0.01

To comprehensively evaluate the editing activity, independent T_0_ transgenic rice plants were regenerated. The *OsMLH1* levels in the ePE5c lines were compared with those in the ePE3 lines via qRT–PCR. Consistent with the findings in calli, the *OsMLH1* abundance in ePE5c plants was significantly lower than that in ePE3 plants (Fig. 2a , *P* < 0.05). The average *OsMLH1* expression in the ePE5c lines was 16.16% to 30.53% of that in the ePE3 lines. At certain targets, e.g., MPK3-T and WRKY32-T, the transcripts of *OsMLH1* in almost half of the ePE5c plants were virtually eliminated to less than 20% of those in ePE3 plants (Additional file 1: Fig. S3), confirming the effectiveness of RNAi inhibition.

Next, precise edits in T_0_ plants were identified via an NGS-based high-throughput tracking of mutations (Hi-TOM) assay [18]. We found that the ePE5c lines had greater or similar frequencies of mutant at the targets, which was consistent with the results obtained from calli (Table 1, Fig. 2b). Detailed analysis revealed that, on average, 87.15% of the regenerated plants were edited by ePE5c, while 71.53% were edited by ePE3. Importantly, the frequency of plants harboring homozygous edits increased from 41.67% for ePE3 to 62.50% for ePE5c at D2-T, from 6.25% to 29.17% at MPK3-T, from 0 to 31.25% at NRT1.1-T, from 4.17% to 16.67% at Pid3-T, from 35.42% to 66.67% at SLR1-T, and from 56.25% to 85.42% at WRKY32-T (Table 1, Fig. 2b), showing obvious increases in the frequency of homozygous mutants resulting from the suppression of *OsMLH1*. In addition, the frequency of ePE5c plants harboring indels was comparable to that of ePE3 plants. These results suggest that ePE5c is superior to ePE3 for conducting precise editing in rice.

**Table 1:** Prime editing of ePE3 and ePE5c in T_0_ transgenic plants

Target	PEs	Examined plants	WT	Mutants (%)	Targeted mutations^a^		Targeted mutations and indels^b^	Indels^c^
					HO (%)	HE		
D2-T	ePE3	48	12	36 (75.00%)	20 (41.67%)	9	7	0
	ePE5c	48	3	45 (93.75%)	30 (62.50%)	12	3	0
MPK3-T	ePE3	48	13	35 (72.92%)	3 (6.25%)	16	10	6
	ePE5c	48	10	38 (79.17%)	14 (29.17%)	14	8	2
NRT1.1-T	ePE3	48	26	22 (45.83%)	0 (0.00%)	3	9	10
	ePE5c	48	11	37 (77.08%)	15 (31.25%)	6	10	3
Pid3-T	ePE3	48	23	25 (52.08%)	2 (4.17%)	23	0	0
	ePE5c	48	13	35 (72.92%)	8 (16.67%)	27	0	0
SLR1-T	ePE3	48	8	40 (83.33%)	17 (35.42%)	11	11	1
	ePE5c	48	0	48 (100%)	32 (66.67%)	14	1	1
WRKY32-T	ePE3	48	0	48 (100%)	27 (56.25%)	21	0	0
	ePE5c	48	0	48 (100%)	41 (85.42%)	7	0	0

The 48 independent lines of the T_0_ generation were genotyped by Hi-TOM analysis with a 10% threshold. The ratios of plants edited at the target region and plants that carried homozygous mutations to total plants were calculated

^a^Plants that carried targeted mutations but not indels; *HO* homozygous mutation, *HE* heterozygous mutation

^b^Plants that simultaneously harbored targeted mutations and byproduct indels

^c^Plants that carried byproduct indels only

### Genome-wide analysis of the off-target effects of ePE5c in rice

High specificity of PE2 and PE3 has been observed in cell lines and plants [19–21]. We wondered whether the off-target effects of ePE5 would increase as a result of the improvement in efficiency. To this end, T_0_ transgenic plants harboring ePE3 and ePE5c constructs targeting D2-T, MPK3-T, and WRKY32-T were selected after 5 weeks of rooting and analyzed via whole-genome sequencing (WGS). For ePE3 or ePE5c, nine homozygous mutants consisting of three independent lines of each pegRNA were sequenced at an approximate 50 × depth. Six plants transformed with the SpCas9 vector were sequenced as controls, and six untransformed wild-type (WT) rice plants were used to filter the background mutations.

The WGS data were used to call single-nucleotide variants (SNVs) and indels according to a standard protocol [22]. To analyze the pegRNA-dependent off-target effects, 1595 potential off-target sites with up to 5 nt mismatches for the three pegRNAs and 740 sites for the three nicking sgRNAs in the reference genome were predicted by Cas-OFFinder [23] (Additional file 1: Table S1). We found that WGS mutations were absent at the off-target sites in the ePE3 and ePE5c plants.

**Fig. 3:**
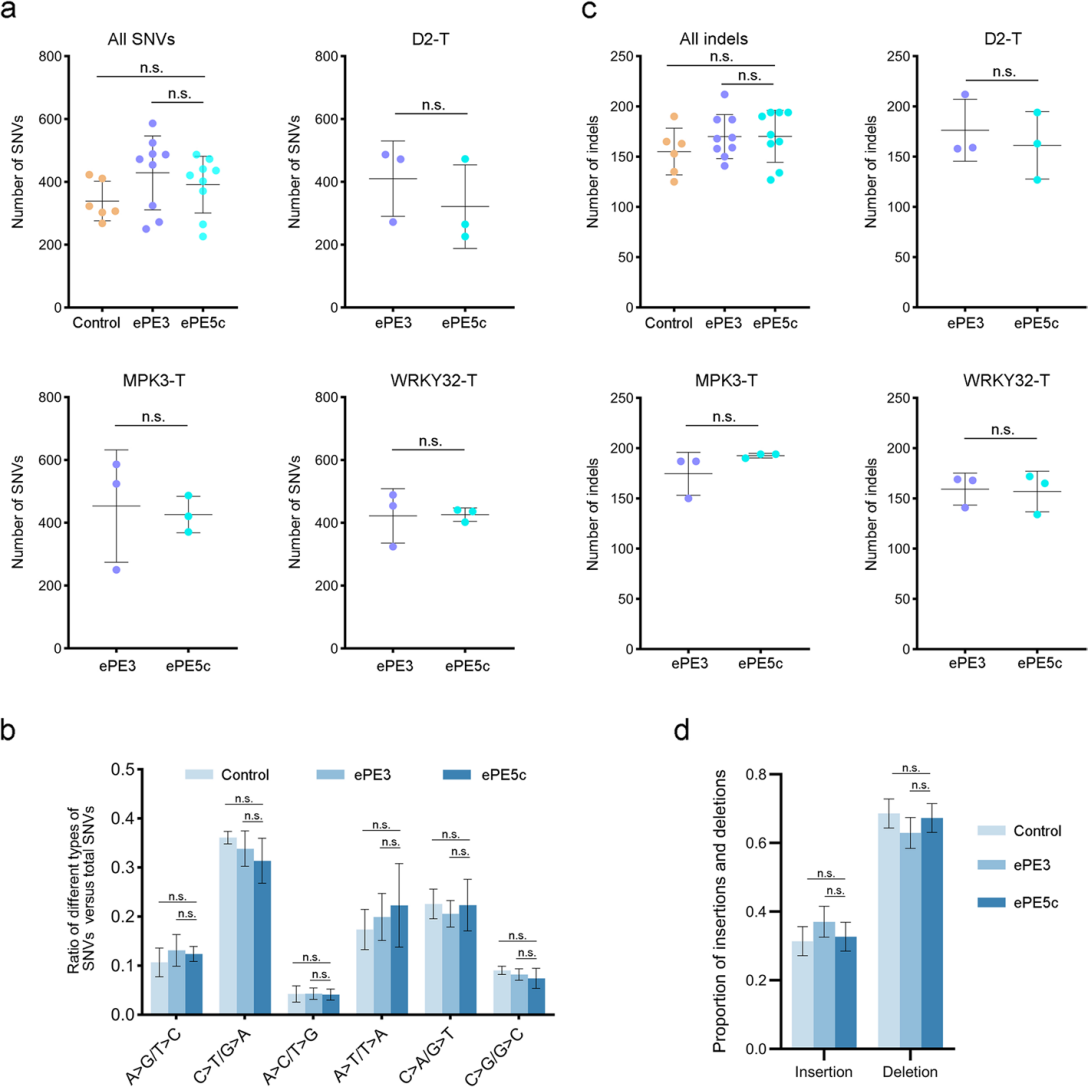
PegRNA-independent off-target analyses of ePE5c by WGS in rice plants. **a** Numbers of SNVs in the control, ePE3, and ePE5c plants. The number of SNVs in each individual line is indicated by a dot. The numbers of SNVs in all the ePE5c plants were compared with those in all the control and ePE3 plants. Alternatively, the numbers of SNVs in D2-T-, MPK3-T-, and WRKY32-T-targeting ePE5c plants were compared with those in ePE3 lines. **b** Comparison of different types of SNVs in ePE5c plants versus control and ePE3 plants. The average numbers were obtained from six SpCas9 control plants and from nine ePE3 and ePE5c plants. **c** Numbers of indels in the control, ePE3, and ePE5c plants. **d** Proportions of insertions and deletions among indels identified in the control, ePE3, and ePE5c plants. The means ± SDs are presented. The differences were assessed by a two-sided Mann–Whitney test. n.s., *P* > 0.05

To evaluate the pegRNA-independent off-target effects, an average of 339, 429, and 391 SNVs per plant were detected in the control, ePE3, and ePE5c groups, respectively. By comparing the numbers of SNVs, we found that ePE5c-induced genome-wide base substitutions were indistinguishable from those observed in the ePE3 and control groups (Fig. 3a, *P* > 0.05). In addition, the preference patterns for the substitution types in ePE5c plants were statistically identical to those in the control and ePE3 plants (Fig. 3b, *P* > 0.05). The SNVs of ePE3 and ePE5c were subsequently mapped to the genome. The random distribution across the chromosomes suggested the absence of mutation hotspots (Additional file 1: Fig. S4). Further annotation of the mutations revealed that the ePE5c lines had similar distributions of SNVs in coding sequences and noncoding regions compared with the ePE3 and control lines (Additional file 1: Fig. S5, *P* > 0.05). In addition, an average of 155, 170, and 170 indels were identified in the control, ePE3, and ePE5c plants, respectively. The overall number and proportion of indels in the ePE5c plants were not different from those in the ePE3 or control plants (Fig. 3c, d; *P* > 0.05). A comparison of the plants according to the target sites also confirmed that ePE5c had a similar number of indels as ePE3 (Fig. 3c; *P* > 0.05). Overall, we could conclude that ePE5c did not induce detectable pegRNA-independent off-target effects in the transgenic plants.

### Reduced fertility of ePE5c plants

It has been reported that *osmlh1*-knockout plants exhibit normal vegetative growth but are sterile or at least partially sterile during the reproductive stage [24–26]. To examine the morphological changes caused by *OsMLH1* RNAi, independent ePE3 and ePE5c homozygous T_0_ mutants of MPK3-T and WRKY32-T were grown in the field for seed production. As expected, the ePE3 and ePE5c lines exhibited regular growth during the vegetative stage, whereas the fertility of the ePE5c plants was markedly lower than that of the ePE3 plants (Fig. 2c, d). The average seed-setting rate of the ePE5c plants was 19.89%, which was significantly lower than that of the ePE3 plants (82.64%) (*P* < 0.05; Fig. 2e). We also observed nearly complete sterility in some plants, e.g., the seed-setting rates were as low as 1.64% in line #6 of ePE5c-MPK3-T and 3.57% in line #1 of ePE5c-WRKY32-T (Additional file 1: Fig. S3). Therefore, although enhanced editing efficiency was obtained, ePE5c may not be an ideal system for heritable genetic engineering due to fertility reduction.

### Conditional excision of the OsMLH1-RNAi cassette in ePE5c T-DNA

**Fig. 4:**
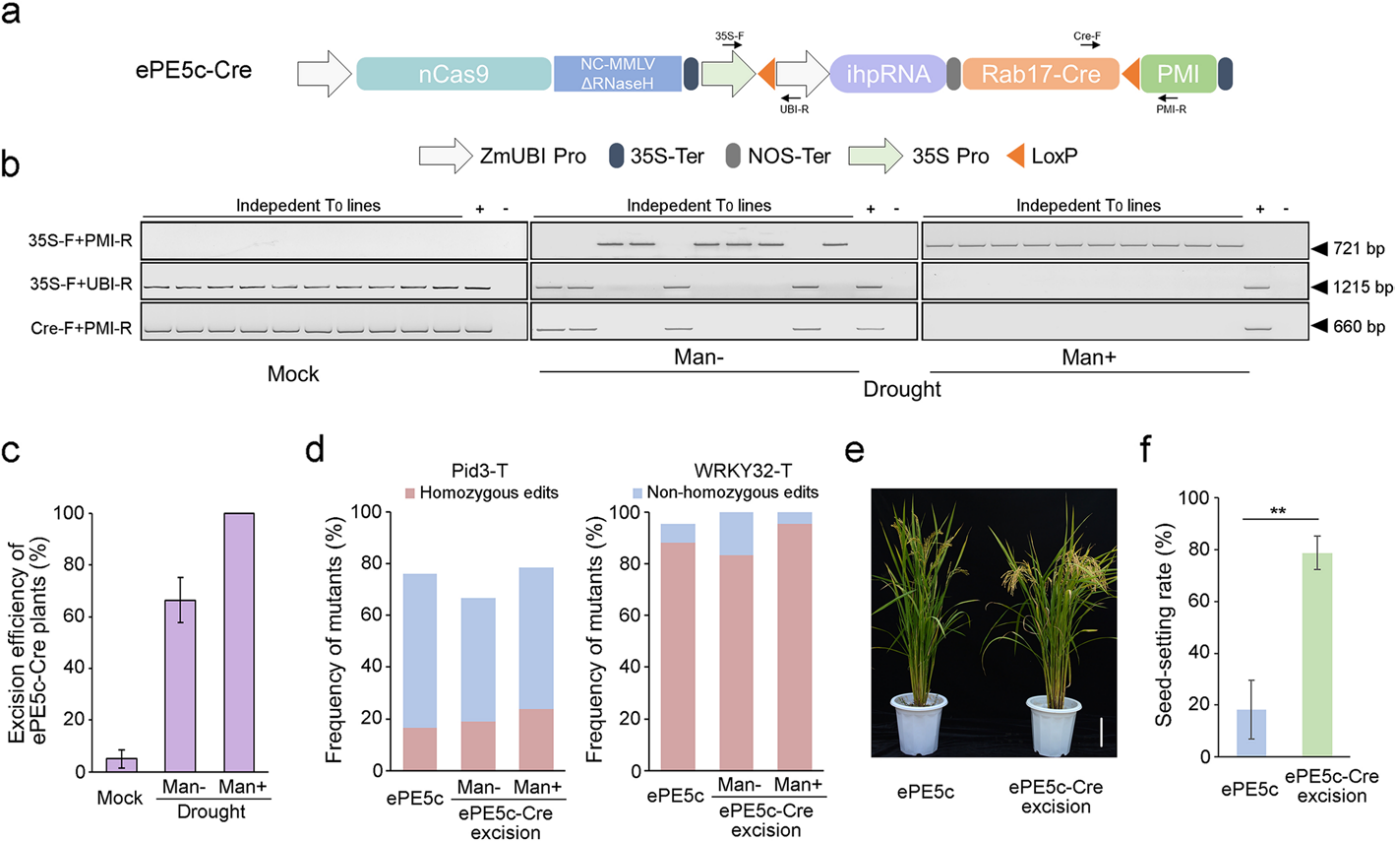
Conditional excision of the *OsMLH1*-ihpRNA cassette during editing. **a** Schematic diagram of the ePE5c-Cre system. 35S Pro, CaMV35S promoter. The black arrows indicate primers used for the identification of excision. **b** Analysis of excision in T_0_ lines. Plants were regenerated from untreated resistant calli (Mock) or from drought-stressed calli with (Drought/Man +) or without (Drought/Man −) additional mannose selection. Representative PCR products from ten ePE5c-Cre-WRKY32-T lines subjected to each treatment are shown. The ePE5c-Cre plasmid was used as a positive control ( +) for amplification, and the genomic DNA of untransformed plants was used as a negative control ( −). **c** Excision efficiency of ePE5c-Cre plants. All regenerated plants listed in Additional file 1: Table S3 were examined via PCR. The excision frequencies of ePE5c-Cre-Pid3-T/WRKY32-T were calculated and averaged to obtain the mean efficiencies. **d** Editing of the excised lines of ePE5c-Cre. The target mutations in Pid3-T (left) and WRKY32-T (right) were examined in 42 excised lines of ePE5c-Cre. The same number of ePE5c lines was tested in parallel as a control. **e** Comparison of the gross morphology of ePE5c lines and excised lines of ePE5c-Cre. Representative ePE5c and excised ePE5c-Cre T_0_ lines harboring homozygous mutations at WRKY32-T are shown at the yellow ripening stage. Excision restored the sterility of ePE5c. Scale bar = 10 cm. **f** Statistical analysis of the seed-setting rates of the ePE5c and ePE5c-Cre lines. The seed-setting rates were determined in the homozygous mutants, which included 16 excision lines of ePE5c-Cre (8 lines for Pid3-T and 8 random lines for WRKY32-T, *n* = 16) and 15 lines of ePE5c (7 lines for Pid3-T and 8 random lines for WRKY32-T, *n* = 15). The differences were assessed by an independent *t* test. **, *P* < 0.01

Given that most heritable edits are produced by the CRISPR system in the early stage of rice callus transformation [27], we hypothesized that eliminating the *OsMLH1* RNAi elements before the regeneration of ePE5c plants might enhance the editing efficiency without disrupting fertility. To remove the *OsMLH1*-ihpRNA cassette from transgenic plants, ePE5c-Cre was designed by integrating a conditional Cre-LoxP recombination system into the ePE5c system. A drought-inducible Rab17 promoter was used to drive Cre, which mediates site-specific DNA recombination between the two LoxP sites in the T-DNA to excise the *OsMLH1*-RNAi element and the Cre expression cassette itself. In addition, a CaMV 35S promoter and a *phosphomannose isomerase* (*PMI*) selectable marker gene were added upstream of the first LoxP site and downstream of the second LoxP site, respectively (Fig. 4a). Once excision occurs, the *PMI* marker should be activated, thus enriching the recombination events under mannose selection.

We examined the editing of ePE5c-Cre at the Pid3-T and WRKY32-T sites in *Agrobacterium*-mediated stable rice transformation. To establish rigorous controls, ePE5c was also used to edit the same targets. After hygromycin selection, resistant ePE5c and ePE5c-Cre calli were obtained at comparable frequencies, suggesting that the Cre system did not inhibit the growth of cells (Additional file 1: Table S2.). Subsequently, the resistant calli were air-dried for 36 h to induce Cre expression. The regeneration rates of drought-treated calli did not decrease and even slightly increased (Additional file 1: Table S3). To enrich the excised products, mannose was added to the regeneration medium. Due to the lack of *PMI* gene, ePE5c calli cannot survive with mannose supplementation. In contrast, plants were regenerated from drought-induced ePE5c-Cre cells, suggesting the activation of *PMI* through successful excision. However, the average regeneration frequency of ePE5c-Cre calli under additional mannose selection decreased to 11.0% compared with the frequency of 22.7% obtained from regular transformation (Additional file 1: Table S3).

Cre-induced recombination in the regenerated plants was examined by three site-specific PCRs (Fig. 4b). A primer for the CaMV 35S promoter (35S-F) and a primer for the *PMI* gene (PMI-R) were designed across the ~ 5.9-Kb Cre and *OsMLH1*-RNAi cassettes to identify the excision site. Two primers were further developed upstream of the *OsMLH1*-RNAi cassette (UBI-R) and downstream of the Cre cassette (Cre-F). The primer pairs 35S-F/UBI-R and Cre-F/PMI-R were also used to verify Cre-mediated recombination. The excision statuses of all the regenerated ePE5c-Cre plants were examined. Even without drought treatment, auto-excision still occurred in 2.4 to 5.6% of the lines (Fig. 4c). This unexpected recombination may be caused by leakage of the Rab17 promoter under non-inducing conditions [28]. After drought exposure, the excision frequency increased to 60.2% in the ePE5c lines targeting the WRKY32-T site and 72.5% in the plants targeting Pid3-T site. During mannose-facilitated regeneration, none of the plants harbored the *OsMLH1*-RNAi and Cre molecular modules (Fig. 4c). These results validated the enrichment of recombination events by the PMI activation strategy. To confirm the precision of recombination, the 35S-F/PMI-R amplicons of several excision lines were analyzed. Sanger sequencing showed that only a copy of LoxP was retained in the T-DNA fragment as designed (Additional file 1: Fig. S6).

### OsMLH1-RNAi excision rescues plant fertility without disrupting the editing of ePE5c-Cre

To elucidate the effect of excision on PE activity, the editing of ePE5c-Cre was assessed in plants from the same batch of transformation. By genotyping independent T_0_ events at the Pid3-T site (Fig. 4d), we found that 28 out of 42 ePE5c-Cre plants (with an occurrence frequency of 66.67%) carried the desired mutation, comparing to the editing of ePE5c in 32 out of 42 lines (76.19% frequency). In addition, 8 lines of ePE5c-Cre and 7 lines of ePE5c were precisely edited at both alleles, resulting in homozygous mutation frequencies of 19.05% and 16.67%, respectively. At the WRKY32-T site, all the ePE5c-Cre plants and 95.24% of the ePE5c plants were edited (Fig. 4d). Among them, 88.10% of the ePE5c lines and 83.33% of the ePE5c-Cre lines were homozygous mutants. Furthermore, the mannose selection-enriched excised lines were also analyzed. At the Pid3-T site, 78.57% of the plants were edited, including 23.81% of the plants with homozygous mutations. In addition, 100% of the plants were edited at the WRKY32-T site, including 95.24% of the plants with homozygous mutations (Fig. 4d). These results demonstrated that the ePE5c-Cre system was remained highly effective in excised lines.

Fertility should be restored after removal of the *OsMLH1*-ihpRNA cassette of ePE5c-Cre. To verify this hypothesis, the reproductive performance of the excised plants was analyzed. Markedly improved fertility was observed in the excised ePE5c-Cre lines compared with the ePE5c lines (Fig. 4e). Additionally, the seed-setting rates of the excised ePE5c-Cre plants were significantly greater than those of the ePE5c plants (Fig. 4f), confirming that ePE5c-induced sterility can be eliminated by conditional excision.

## Discussion

DNA MMR strongly antagonizes the PE of base substitutions and small indels [9, 10]. The PE efficiency can be reliably enhanced by the expression of *hMLH1dn* to transiently ablate MMR activity in human cells [9]. However, strategies for expressing *hMLH1dn* or its plant orthologs have been less exploited in extensive studies of engineered plant prime editors [29–34]. Limited data from MLH1dn-faciliated plant prime editors have shown vague and inconsistent effects on efficiency, although the percentage of homozygous mutants could increase at some targets, possibly due to long-term expression. In this report, we provide further data showing that neither exogenous *hMLH1dn* nor endogenous *OsMLH1dn* significantly improved the editing activity of our recently optimized ePE2 system in stably transformed rice cells. Given the markedly lower number of exogenous gene copies delivered during *Agrobacterium*-mediated plant transformation than during mammalian cell transient transformation, it is reasonable to suspect that the expression of *MLH1dn* in plant prime editors may be insufficient to effectively inhibit MMR. In addition to the use of dominant-negative variants, the editing efficiency could be enhanced by direct manipulation of the *MLH1* transcript via CRISPRi or siRNA in HEK293 or K562 cells [9, 10]. Inspired by these results, we attempted to optimize the ePE2 system through the disruption of *MLH1* accumulation via RNAi in plants. The resulting ePE5c system exhibited significantly increased editing efficiency and yielded obviously increased ratios of both overall mutants and homozygous mutants. We believe that this approach can be easily adopted for other species by integrating the RNAi module of native *MLH1* or other MMR genes into prime editors and thus constitutes a stable and conspicuous strategy to improve plant PE efficiency.

Loss-of-function *mlh1* mutants of monocots and dicots consistently exhibit reproductive defects [24–26, 35]. In rice, the seed-setting rate of loss-of-function lines could be reduced to approximately 40% in *indica* Guangluai4 background and to less than 20% in *japonica* Dongjing [26]. Moreover, the average rates decreased to less than 10% in knockout mutants of *indica* Huazhan [24], which is similar to the approximate 80–90% reductions observed in CRISPR-induced mutants of *japonica* ZH11 [25]. In this study, the RNAi-mediated suppression of *OsMLH1* led to severe reproductive disturbances in *japonica* Nipponbare plants as well. Therefore, the RNAi module of ePE5c presumably hampers the fertility of various rice varieties, which largely diluted its benefits on efficiency enhancement. Although the *OsMLH1*-ihpRNA cassette-containing T-DNA could be segregated out in T_0_ progenies by self-crossing (Additional file 1: Table S4), the small number of seeds notably impedes the screening of T-DNA-free edited offspring. To solve this problem, a conditional Cre-mediated excision system was introduced into the ePE5c vector [36]. Simple and short-term drought treatment induced precise excision in 66.4% of the transgenic plants, restoring the fertility of the plants with enhanced PEs to normal. With an enrichment system through simple selection, 100% excision could be achieved, which would greatly reduce the screening cost of excised plants. Interestingly, the regeneration frequency of ePE5c-Cre decreased under double selection with hygromycin plus mannose, whereas a minor increase in the homozygous mutation ratio was observed. A possible reason for this finding is that plants were over-selected by additional pressure, resulting in mild retardation of growth as well as enrichment of cells with high expression. Consequently, normal regeneration and a high proportion of excision could be expected when using mannose as the sole selection agent during the regeneration process in the future.

Failure of MMR may impair the maintenance of the plant genome. Artificial expression of dominant-negative variants of AtPMS2, which dimerizes with MLH1 to form a MutLα heterodimer, or OsPMS1, which dimerizes with MLH1 to form a MutLβ heterodimer, increased the base substitution ratio and induced microsatellite instability (MSI) of genome [37, 38]. MSH2, the conserved protein of the MutS complex, has been proven to be involved in the regulation of somatic mutations and homologous recombination (HR) in *Arabidopsis* and tobacco [39–41]. A Tos17 insertion mutation in the rice MutSα component *OsMSH6* resulted in the accumulation of spontaneous single-nucleotide mutations, HR, and MSI [42, 43]. Although there is a lack of direct evidence of the effect of MLH1 on the mutation burden of plants, stable tissue-specific suppression or the null mutation *mlh1* increased the MSI in mice [44, 45]. Therefore, transient but not stable MMR inhibition during PE is desirable to avoid potential damage to the plant genome. A previous study showed that 3 days of PE4 expression did not trigger MSI in HeLa cells [9]. In addition, no *OsMLH1dn*-associated random mutations were found in the progeny of transgenic rice of PE5max [46]. Our results confirmed that ePE5c did not significantly increase the frequency of whole-genome base mutations in newly regenerated T_0_ rice. However, we cannot rule out the possibility of genome damage by whole-growth-period expression of ePE5c. To alleviate this risk, an excision system was designed to remove the *OsMLH1*-ihpRNA cassette after 3 weeks of selection. Effective editing of ePE5c may occur much earlier than plant regeneration; therefore, excision does not attenuate the efficiency improvement resulted from *OsMLH1* suppression. Considering that drought treatment is difficult during callus growth on medium, the Rab17 promoter of ePE5c-Cre can be replaced with a heat- or light-induction system to activate recombination. In this case, excision would be conveniently induced at a series of time points. By comparing the editing efficiencies, a minimal time requirement of RNAi suppression can be determined to maximally reduce the risk of genome damage while maintaining a promising enhancement effect. During the preparation of this manuscript, David Liu’s group published a report showing that transient inhibition of MMR through microinjection of mMLH1dn mRNA into two-cell-stage mouse embryos resulted in highly efficient prime editing [47]. WGS of the edited mice also revealed that transient inhibition of *MLH1* resulted in identical SNVs and a limited increase in the number of indels in repetitive sequences, which did not lead to any detectable phenotypic consequences [47]. These data also confirmed our hypothesis in plants that short-term inhibition of MMR genes can enhance PE efficiency with a low risk of genetic instability for research or breeding purposes. Taken together, the results of this proof-of-concept study indicate that prime editor optimization via an excisable RNAi module of MMR genes is an attractive strategy for substantially improving plant editing efficiency in a safe and reliable manner.

## Conclusions

The highly specific ePE5c system significantly improved PE efficiency in stably transformed rice calli and regenerated plants through direct RNAi knockdown of *OsMLH1* instead of coexpressing MLH1dn variants. The knockdown of *OsMLH1* led to a reduction in fertility in ePE5c plants, which could be overcome by conditional excision of the RNAi module without hindering the improvement of activity. This optimization by transient MMR inhibition could substantially enhance the efficiency without disturbing plant development, providing a safe and reliable strategy that can be widely applied in engineering efficient PE systems for different plant species.

## Methods

### Vector construction

All the vectors were developed from the pHUC-ePE2 backbone [16]. To construct ePE2-hMLH1dn for the ePE5a system and ePE2-OsMLH1dn for the ePE5b system, codon-optimized hMLH1dn [15] and OsMLH1dn [13] were added to the 3′ terminus of ePE2 with a P2A self-cleavage linker. To construct the ePE5c system, a 266-bp coding sequence spanning exons 2, 3, and 4 of *OsMLH1* was selected as the RNAi region (Additional file 1: Fig. S1). The sequence and its reverse repeat were amplified from cDNA and inserted into opposite ends of an intron from potato *GA20ox1-3* [48]. The constructed ihpRNA was subsequently introduced downstream of the maize ubiquitin 1 promoter. The RNAi cassette was then subcloned and inserted into ePE2 via Pme I digestion. The PE3 and PE5 strategies were performed by assembling the OsU3-driven sgRNA cassette into the ePE vectors as previously described [49].

To establish the conditional excision system, the Rab17 promoter was cloned from maize genomic DNA. The plant codon-optimized coding sequence of Cre was separated with an intron of the *Arabidopsis* endo-1,4-beta-D-glucanase gene *KOR* to minimize unintended expression. A terminator from the pea *rbcS* gene was linked downstream of the synthesized Cre gene to form the expression cassette (Genscript, Nanjing, China). The LoxP-RNAi-Cre-LoxP module was constructed via Gibson assembly. To enrich the excision events, we replaced the *OsMLH1*-ihpRNA cassette with the *PMI* cassette in ePE5c. The excision module was subsequently inserted between the CaMV 35S promoter and *PMI* [50] to construct ePE5c-Cre. All the vectors were verified by Sanger sequencing (Sangon, Shanghai, China).

The design and integration of gRNAs were conducted according to previous methods [15]. To provide negative selection of recombinant plasmids, the resistance genes for spectinomycin and gentamicin were pre-integrated into the pegRNA and nicking sgRNA cassette, respectively. The clones were screened by bacterial PCR and confirmed by Sanger sequencing. The high-fidelity polymerase, restriction endonucleases, ligases, and Gibson mixtures used in the study were obtained from NEB (Ipswich, USA). The pegRNA sequences and sgRNA nicking sites are listed in Additional file 1: Table S5. The detail of conditional excision RNAi module is shown in the Additional file 1: Supplemental Sequence.

### Rice transformation and plant growth

Embryonic calli were induced from mature seeds of rice (*Oryza sativa* spp. *japonica* cultivar Nipponbare) for 2 to 3 weeks. Well-grown calli were collected and transformed with *Agrobacterium* strain EHA105. The infected calli were selected with 50 mg/L hygromycin. The emergence of calli from one initial callus was recognized as an independent event. After 3 to 4 weeks of selection, three yellowish, spherical calli from each resistance event were transferred to regenerate plants under 25 mg/L hygromycin selection. When the regenerated plants had grown to a length of 2–3 cm, they were transferred to rooting media for another 4 to 5 weeks. For the assessment of fertility, the genotyped plants were grown from May to October in the field at Hefei (32° 28′ 48″ N, 117° 10′ 12″ E) or in a greenhouse with a 16 h/8 h photoperiod at 26 °C to 30 °C.

The conditional excisions were guided based on a previously described strategy [36]. The resistant calli were desiccated on a stack of three dry 90-mm filter papers in Petri dishes for 36 h. The treatment was conducted at 28 °C in the dark to maintain differentiation activity. For the enrichment of excised plants, 5 g/L sucrose + 15 g/L mannose was used as the carbon source in the regeneration medium to select PMI-resistant plants [50].

### Analysis of on-target editing

To determine the on-target editing efficiencies of PE systems, newly emerged calli were sampled after 2 to 3 weeks of selection. From each transformant, approximately 200 calli with diameters less than 4 mm were collected and ground together. A commercial plant genomic DNA isolation kit was used for the extraction and purification of genomic DNA (Tiangen Biotech, Beijing, China). The amplicons were amplified with Q5 high-fidelity polymerase and sequenced on the Illumina NextSeq platform via a paired-end 150-bp (PE-150) strategy (Genwiz, Suzhou, China). More than 0.5 Gb of data were generated per sample and analyzed with CRISPResso2 software [51]. The amplicon-NGS data have been deposited in the SRA database under the BioProject accession number PRJNA1073312 [52].

To identify edits in plants, leaves of independent lines were collected from at least three different tillers for DNA extraction. The targeting sites were examined by Hi-TOM sequencing with a 10% threshold. The screened mutations were randomly verified by Sanger sequencing PCR amplicons. All primers used in this study are listed in Additional file 1: Table S5.

To genotype the progenies, seeds of independent T_0_ lines were dehulled, sterilized, and germinated in 1/2 MS medium for a week. Targeted editing was confirmed by Hi-TOM analysis. Two pairs of primers for the RNAi module and the Cas9 module were designed to amplify the T-DNA fragment.

### WGS analyses

Six WTs, six SpCas9 transgenic T_0_ lines, nine ePE3 T_0_ homozygous mutants (consisting of three lines for each target), and nine ePE5c homozygous mutants were subjected to WGS. Genomic DNA was extracted from the leaves of the plants after 5 weeks of rooting using a DNA isolation kit (Tiangen). The quality of the extracted DNA was inspected, after which the DNA was subsequently used to construct DNA libraries. Rice genome sequencing was carried out on the DNB SEQ platform (BGI, Shenzheng, China).

Each sample yielded an average of approximately 20 Gb of clean sequencing reads, with an average depth of 50 × . The original sequencing data files obtained from the platform were subjected to base recognition analysis, after which the sequences were transformed into raw sequences. These results were stored in the FASTQ file format. To ensure data quality, fastp software (v0.20.1) was used for quality control and to filter the raw reads [53], resulting in the acquisition of clean reads. All subsequent analyses were based on these clean reads.

The clean reads were subsequently mapped to the Nipponbare genome using the BWA software (v0.7.17). The resulting sequence alignment/MAP (SAM) files were converted to binary alignment/map (BAM) files using SAMtools with the “sort” parameter [54]. Next, SAMtools (with the “markdup-r” parameter) was used to remove duplicates from the sorted comparison results for downstream analysis. The variation in the samples was then determined using GATK (version 4.2.5.0) with reference to the file with the comparison results. To identify SNVs, overlaps were determined using three mutation identification software packages: GATK (v4.2.5.0), LoFreq (v2.1.2), and Strelka2 (v2.9.10) [55–57]. Similarly, the GATK (V4.2.5.0) and Strelka2 (v2.9.10) software packages were used for calling indels. Background mutations from the wild-type plants were filtered out, resulting in the final set of SNVs and indels.

To ensure data quality, the quantity of data from each individual sample was required to be no less than 90% of the target data volume. Additionally, the sequencing coverage of all the samples had to reach 99%. The number of clean reads obtained exceeded 1.4 × 10^9^, and the mapping rate reached 99.98%.

### Identification of excisions

The excision of all the regenerated ePE5c-Cre lines was assessed by PCR with the 35S-F, UBI-R, Cre-F, and PMI-R primers (Additional file 1: Table S5). To confirm precise recombination, the amplicons of 35S-F and PMI-R from 12 excised lines were subjected to Sanger sequencing. To determine the effects of the Cre system on editing, 42 independent T_0_ lines from each treatment were genotyped via the Hi-TOM assay.

### Reverse transcription and quantitative PCR

Independent callus events (~ 0.1 g per sample) after 3 weeks of selection or leaves (~ 0.2 g per sample) of regenerated T_0_ lines after 5 weeks of rooting were used for the extraction of total RNA using an RNA Easy Fast Plant Tissue Kit (Tiangen Biotech). A FastKing RT Kit was used for reverse transcription of cDNA, which was used as the template (Tiangen Biotech). qRT–PCR analysis was performed with a QTOWER 2.0 system (Analytik Jena, Germany). The expression of *OsMLH1* was quantified with the ∆∆Ct method using *OsACTIN2* as the reference housekeeping gene.

### Supplementary Information

Additional file 1: Fig. S1. Diagram of the RNAi construct of *OsMLH1*. Fig. S2. Byproduct indel efficiencies of ePE3 and ePE5c in calli. Fig. S3. Editing of ePE3 and ePE5c in individual transgenic plants. Table S1. Putative pegRNA spacer or nick sgRNA sequence-like off-target sites predicted by Cas-OFFinder. Fig. S4. Genomic distribution of the SNVs and indels identified by WGS. Fig. S5. Numbers of SNVs located in different gene regions. Table S2. Selection of calli transformed with ePE5c and ePE5c-Cre under hygromycin pressure. Table S3. Plant regeneration of ePE5c and ePE5c-Cre transformants. Fig. S6. Sequencing of the excision site of ePE5c-Cre plants. Table S4. Transmission of edits and T-DNA in the T_1_ generation of ePE5c. Table S5. Sequences of the pegRNAs, nicking sgRNAs, and primers used in this study. Supplemental Sequence.

Additional file 2. Review history.

## Data Availability

All data supporting the findings of this study are available in the article and its supplementary figures and tables. The amplicon-NGS data can be achieved in the SRA database under the BioProject accession number PRJNA1073312 [52].
