# Supplementary material for: Conditional knockdown of OsMLH1 to improve plant prime editing systems without disturbing fertility in rice
Source: Genome Biol. 2024 May 21;25:131. doi: 10.1186/s13059-024-03282-y (PMC11110357; doi:10.1186/s13059-024-03282-y)
Supplement: Supplementary file 1 — Additional file 1: Fig. S1. Diagram of the RNAi construct of OsMLH1. Fig. S2. Byproduct indel efficiencies of ePE3 and ePE5c in calli. Fig. S3. Editing of ePE3 and ePE5c in individual transgenic plants. Table S1. Putative pegRNA spacer or nick sgRNA sequence-like off-target sites predicted by Cas-OFFinder. Fig. S4. Genomic distribution of the SNVs and indels identified by WGS. Fig. S5. Numbers of SNVs located in different gene regions. Table S2. Selection of calli transformed with ePE5c and ePE5c-Cre under hygromycin pressure. Table S3. Plant regeneration of ePE5c and ePE5c-Cre transformants. Fig. S6. Sequencing of the excision site of ePE5c-Cre plants. Table S4. Transmission of edits and T-DNA in the T1 generation of ePE5c. Table S5. Sequences of the pegRNAs, nicking sgRNAs, and primers used in this study. Supplemental Sequence. [file 13059_2024_3282_MOESM1_ESM.pdf]

**Additional File 1**

1. **Fig. S1.** Diagram of the RNAi construct of *OsMLH1*.

2. **Fig. S2.** Byproduct indel efficiencies of ePE3 and ePE5c in calli.

3. **Fig. S3.** Editing of ePE3 and ePE5c in individual transgenic plants.

4. **Table S1.** Putative pegRNA spacer or nick sgRNA sequence-like off-target sites predicted by Cas-OFFinder.

5. **Fig. S4.** Genomic distribution of the SNVs and indels identified by WGS.

6. **Fig. S5.** Numbers of SNVs located in different gene regions.

7. **Table S2.** Selection of calli transformed with ePE5c and ePE5c-Cre under hygromycin pressure.

8. **Table S3.** Plant regeneration of ePE5c and ePE5c-Cre transformants.

9. **Fig. S6.** Sequencing of the excision site of ePE5c-Cre plants.

10. **Table S4.** Transmission of edits and T-DNA in the $T_1$ generation of ePE5c.

11. **Table S5.** Sequences of the pegRNAs, nicking sgRNAs, and primers used in this study.

12. **Supplemental Sequence.**

Fig. S1

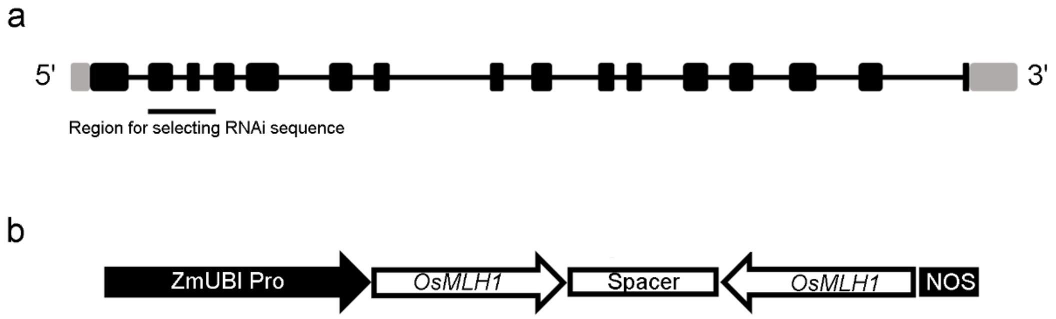


**Fig. S1.** Diagram of the RNAi construct of *OsMLH1*.

(a) Gene structure of *OsMLH1.* The exons are indicated by black boxes, the introns are indicated by black boxes, and the UTRs are indicated by gray boxes. The exon sequence of the underlined regions was selected for RNAi of *OsMLH1.* (b) Schematic illustrations of the ihpRNA cassette used for RNAi. The sense and antisense coding sequences of the underlined region in the panel (a) were separated by an unrelated intron spacer to form a hairpin, which was expressed with the maize ubiquitin 1 promoter and NOS terminator.

Fig. S2

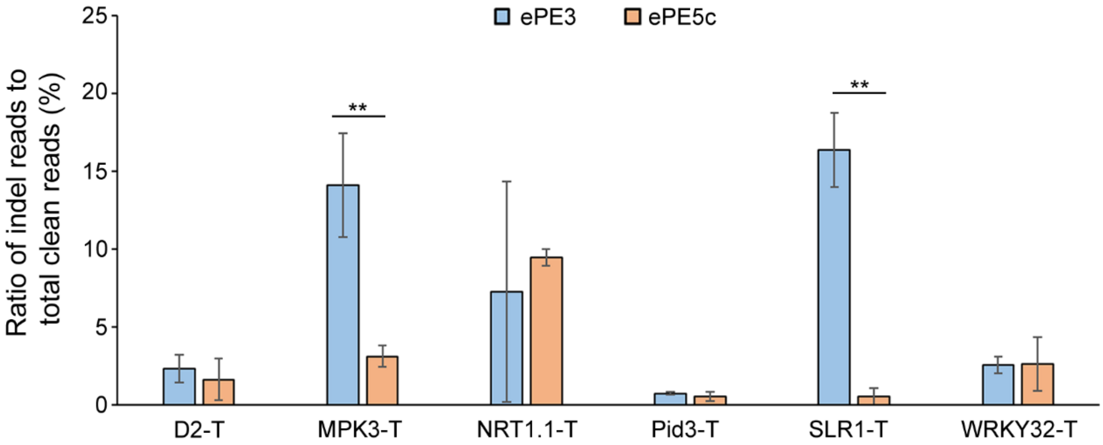


**Fig. S2.** Byproduct indel efficiencies of ePE3 and ePE5c in calli.

The indels in stably transformed calli were identified via amplicon NGS. The efficiencies were calculated from the ratios of edited reads to total clean reads. The mean values and standard deviations of three biological replicates are shown. Paired t tests were used to assess the significance of the differences. *, $P<0.05$; **, $P<0.01$.

Fig. S3

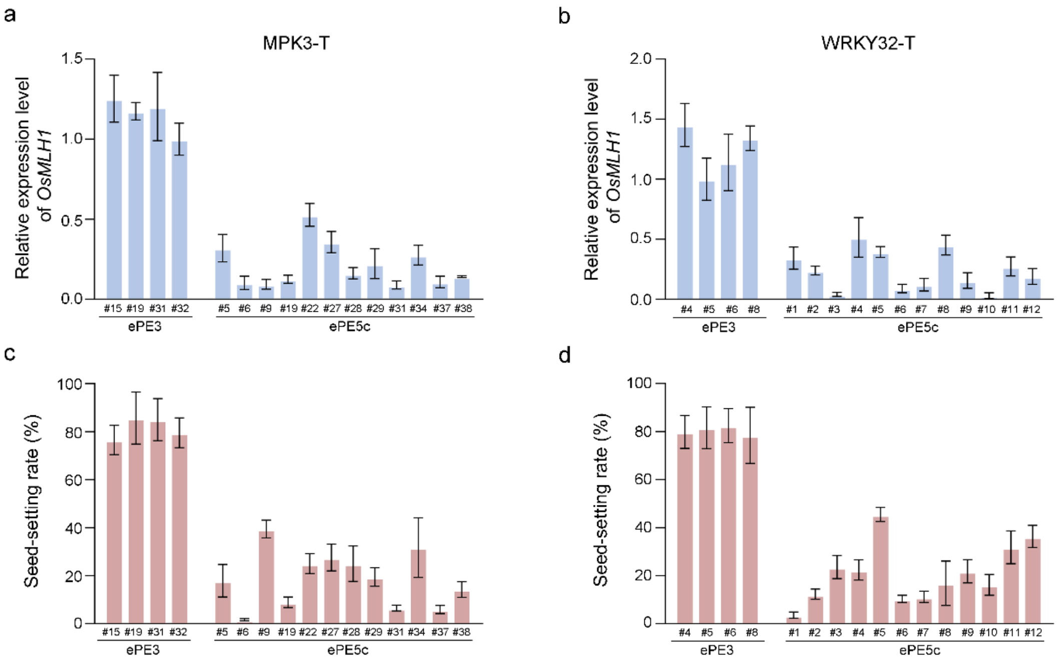


**Fig. S3.** Editing of ePE3 and ePE5c in individual transgenic plants.

The relative expression levels of *OsMLH1* in four ePE3 lines and 12 ePE5c lines targeting MPK3-T (a) and WRKY32-T (b) were measured by qRT–PCR. The mean values and standard deviations were obtained from three technical replicates. The seed setting rates of the corresponding lines are indicated in (c) and (d). For each line, at least three panicles were counted and averaged to obtain the mean frequency.

**Table S1.** Putative pegRNA spacer or nick sgRNA sequence-like off-target sites predicted by Cas-OFFinder.

| Target | 1-nt mismatch | 2-nt mismatches | 3-nt mismatches | 4-nt mismatches | 5-nt mismatches |
|---|---|---|---|---|---|
| *D2*-T | 0 | 2 | 143 | 156 | 658 |
| *D2*-nick | 0 | 1 | 6 | 27 | 229 |
| *MPK3*-T | 0 | 1 | 2 | 47 | 399 |
| *MPK3*-nick | 0 | 1 | 4 | 48 | 273 |
| *WRKY32*-T | 0 | 0 | 1 | 19 | 167 |
| *WRKY32*-nick | 0 | 0 | 0 | 17 | 134 |

Fig. S4

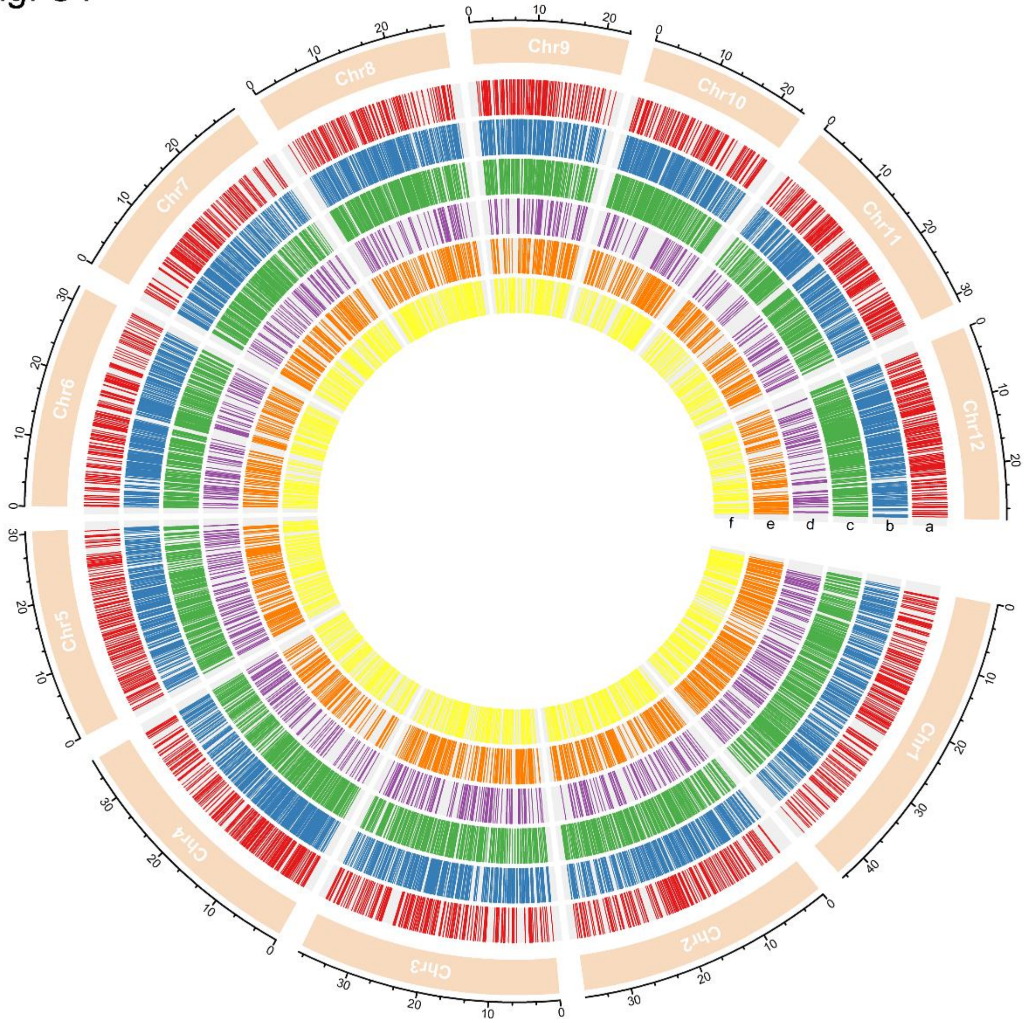


**Fig. S4.** Genomic distribution of the SNVs and indels identified by WGS.

The SNVs (a to c) and indels (d to f) are randomly distributed on the 12 chromosomes in the SpCas9 control (a, d), ePE3 (b, e), and ePE5c (c, f) plants.

Fig. S5

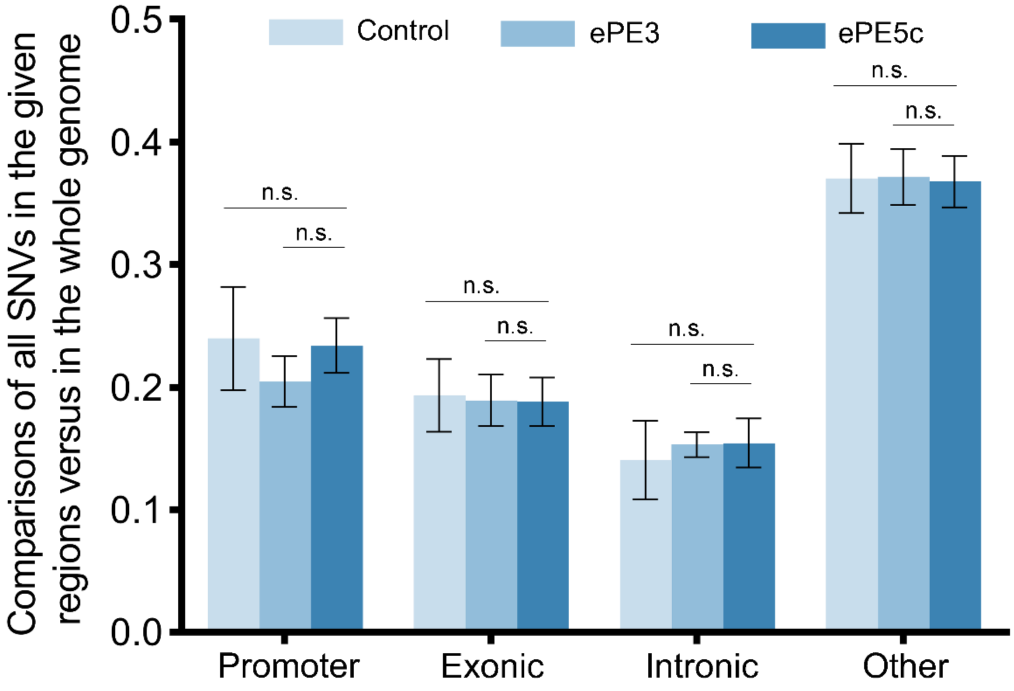


**Fig. S5.** Numbers of SNVs located in different gene regions.

The numbers of SNVs located at the promoter, exonic, intronic and remaining regions of the genome were assayed in individual plants. The means ± SDs were obtained from six SpCas9 control plants, nine ePE3 plants and nine ePE5c plants. Differences were assessed by two-sided Mann–Whitney tests. ns, $P>0.05$.

**Table S2.** Selection of calli transformed with ePE5c and ePE5c-Cre under hygromycin pressure.

| PE | Target | Repeats | Infected calli | Resistant events* | Ratio (%) |
|---|---|---|---|---|---|
| ePE5c | Pid3-T | #1 | 500 | 460 | 92% |
| | | #2 | 500 | 445 | 89% |
| | | #3 | 500 | 385 | 77% |
| | WRKY32-T | #1 | 500 | 415 | 83% |
| | | #2 | 500 | 410 | 82% |
| | | #3 | 500 | 425 | 85% |
| ePE5c-Cre | Pid3-T | #1 | 500 | 390 | 78% |
| | | #2 | 500 | 395 | 79% |
| | | #3 | 500 | 410 | 82% |
| | WRKY32-T | #1 | 500 | 430 | 86% |
| | | #2 | 500 | 370 | 74% |
| | | #3 | 500 | 425 | 85% |

*, new calli grown from a single callus after three to four weeks of selection were considered one independent resistance event.

**Table S3.** Plant regeneration of ePE5c and ePE5c-Cre transformants.

| PE | Treatment | Target | Resistant events* | Regenerated lines# | Ratio (%) |
|---|---|---|---|---|---|
| ePE5c | Mock | Pid3-T | 460 | 112 | 24.35% |
| | | WRKY32-T | 415 | 79 | 19.04% |
| | Drought Man- | Pid3-T | 445 | 76 | 17.08% |
| | | WRKY32-T | 410 | 92 | 22.44% |
| | Drought Man+ | Pid3-T | 385 | 0 | 0 |
| | | WRKY32-T | 425 | 0 | 0 |
| ePE5c-Cre | Mock | Pid3-T | 390 | 66 | 16.92% |
| | | WRKY32-T | 430 | 82 | 19.07% |
| | Drought Man- | Pid3-T | 395 | 80 | 20.25% |
| | | WRKY32-T | 370 | 93 | 25.14% |
| | Drought Man+ | Pid3-T | 410 | 50 | 12.20% |
| | | WRKY32-T | 425 | 42 | 9.88% |

*, the resistant calli shown in Table S2 were used. For each resistance event, three calli were transferred together to generate rice plants for 4 to 5 weeks. #, several plants may be regenerated from one event, and only one plant was selected as the regenerated line.

Fig. S6

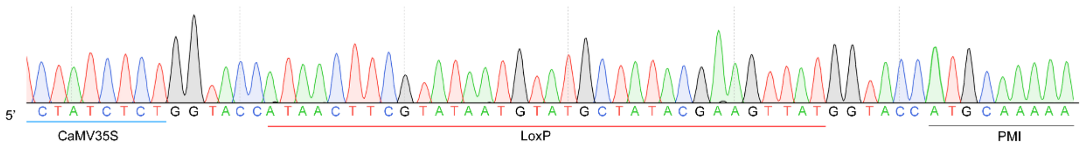


**Fig. S6.** Sequencing of the excision site of ePE5c-Cre plants.

Representative Sanger sequencing chromatogram of the recombination site in excised plants. The precise recombination is shown by the underlined sequences indicating CaMV 35S, LoxP, and *PMI*.

**Table S4.** Transmission of edits and T-DNA in the $T_1$ generation of ePE5c.

| $T_0$ line No.* | Number of $T_1$ plants | Edits[#] | T-DNA[&] |
|---|---|---|---|
| #2 | 16 | 16HO | 14+:2- |
| #3 | 10 | 10HO | 10+ |
| #4 | 16 | 16HO | 16+ |
| #5 | 14 | 14HO | 12+:2- |

*, Self-crossing seeds of four $T_0$ ePE5c lines carrying homozygous edits at the WRKY32-T site were used for progeny genotyping.

[#], The targeted edits were assessed by Hi-TOM analysis with a 10% threshold. The results revealed that all $T_1$ lines harbored homozygous edits (HOs).

[&], Two pairs of primers were designed to detect T-DNA segregation in the $T_1$ generation. The numbers of T-DNA-positive (+) and T-DNA-negative (-) plants are indicated.

**Table S5.** Sequences of the pegRNAs, nicking sgRNAs, and primers used in this study.

1. PegRNA sequences used in the study

| Target | pegRNA Sequence (5' to 3') |
|---|---|
| D2-T | CCCCGCCGAGGACTCCGTCCGTTTCAGAGCTATGCTGGAAACA<br>GCATAGCAAGTTGAAATAAGGCTAGTCCGTTATCAACTTGAAAAA<br>GTGGCACCGAGTCGGTGCGATGAGCACCGTGACGGAGTCCTAA<br>ATATATTTGACGCGGTTCTATCTAGTTACGCGTTAAACCAACTAGA<br>AA |
| MPK3-T | GTCGGAGAGCGACATGATGAGTTTCAGAGCTATGCTGGAAACAG<br>CATAGCAAGTTGAAATAAGGCTAGTCCGTTATCAACTTGAAAAAGT<br>GGCACCGAGTCGGTGCGTGACCACGTCCTCCGTCATCATGTCG<br>CTAAATAATTTTGACGCGGTTCTATCTAGTTACGCGTTAAACCAAC<br>TAGAAA |
| NRT1.1-T | CTCCACTTGCTCGCCGTCGCGTTTCAGAGCTATGCTGGAAACAG<br>CATAGCAAGTTGAAATAAGGCTAGTCCGTTATCAACTTGAAAAAGT<br>GGCACCGAGTCGGTGCGGAGTCGCCGCGACGGCGAGCCTTAC<br>AAATTGACGCGGTTCTATCTAGTTACGCGTTAAACCAACTAGAAA |
| Pid3-T | CTGACTCTGTACTCTTCAAGGTTTCAGAGCTATGCTGGAAACAGC<br>ATAGCAAGTTGAAATAAGGCTAGTCCGTTATCAACTTGAAAAAGT<br>GGCACCGAGTCGGTGCATCCTCCCTCTTAGAAGAGTACAGCCTA<br>AATTTTGACGCGGTTCTATCTAGTTACGCGTTAAACCAACTAGAAA |
| SLR1-T | ACCCCTCGGACCTCTCCTCCGTTTCAGAGCTATGCTGGAAACAG<br>CATAGCAAGTTGAAATAAGGCTAGTCCGTTATCAACTTGAAAAAGT<br>GGCACCGAGTCGGTGCTCTCGACCCAGGTGGAGAGGTCATTTA<br>AACTTGACGCGGTTCTATCTAGTTACGCGTTAAACCAACTAGAAA |
| WRKY32-T | TACACAAGATTACCAGAAAGGTTTCAGAGCTATGCTGGAAACAGC<br>ATAGCAAGTTGAAATAAGGCTAGTCCGTTATCAACTTGAAAAAGT<br>GGCACCGAGTCGGTGCTGCTCCTGGCCTGCTTTCTGGTAATCTC<br>TCTCACATTGACGCGGTTCTATCTAGTTACGCGTTAAACCAACTA<br>GAAA |

2. Nicking sgRNA sequences used in the study

| Target | nicking sgRNA Sequence (5' to 3') |
|---|---|
| D2-nick | GCACTCGCTGAGGAACTTGA |
| MPK3-nick | CTCCGGCGCGCGGTACCACC |
| NRT1.1-nick | CACGAGGACGGACGTGGAGG |
| Pid3-nick | CCCACACACTGACCACCATG |
| SLR1-nick | GCTGGCGGGATAGGGGGCAG |
| WRKY32-nick | TATGGCAGCTCTCTTCTTCT |

3. Primers for Hi-TOM analysis of the targets

| Target | Sequence (5' to 3') |
|---|---|
| D2-T | FP: ggagtgagtacggtgtgcCACCGACGAGCTCATCTCCG |
| | RP: gagttggatgctggatggTATACAGGCTTACTTCCAGT |
| MPK3-T | FP: ggagtgagtacggtgtgcAACGCCAACTGCGACCTCAAGA |
| | RP: gagttggatgctggatggGGCGGAGTAGTCGGTGGAGTTG |
| NRT1.1-T | FP: ggagtgagtacggtgtgcATGCGGCGATCATCGACAGG |
| | RP: gagttggatgctggatggTGGTCGCCCAGATGGGGAGC |
| Pid3-T | FP: ggagtgagtacggtgtgcATCGATGGCGGGAAGGAGGA |
| | RP: gagttggatgctggatggATAGCATTGTAAACATTAGC |
| SLR1-T | FP: ggagtgagtacggtgtgcATGACGGGTTCGTGTCGCACCT |
| | RP: gagttggatgctggatggCAGTGGACGAGGTGGAAGCA |
| WRKY32-T | FP: ggagtgagtacggtgtgcTTAGGATTTAAGACCGTTCC |
| | RP: gagttggatgctggatggGAGAGATCTATGGCAGCTCT |

4. Primers for amplicon deep sequencing

| Target | Sequence (5’ to 3’) |
|---|---|
| D2-T | FP: GACGTGCTCATCGGAGACGGCA<br>RP: AGGTATACAGGCTTACTTCCAG |
| MPK3-T | FP: AACCTGCTGCTGAACGCCAACT<br>RP: TGAGCTCCATGAAGATGCAGCC |
| NRT1.1-T | FP: TGGTTTGTAATATGGGTGCAGG<br>RP: CGTGGATGGTCCAGAACATGAT |
| Pid3-T | FP: TATGATCTGAAGGGCGTCGA<br>RP: CAAAGTCAGCCTTGATAGCA |
| SLR1-T | FP: GGATGGGCGGCGTGAGCGCC<br>RP: GAGTTCAAAGAAGCCGCTAC |
| WRKY32-T | FP: ACGTCTTAGACACCCAGACATT<br>RP: TTCTTCTGGCCGTCTTCTCTCT |

5. Primers for qRT–PCR

| Gene | Sequence (5’ to 3’) |
|---|---|
| *OsACTIN2* | FP: CCTGACGGAGCGTGGTTAC<br>RP: CCAGGGCGATGTAGGAAAGC |
| *OsMLH1* | FP: AGGAGTGTCTATGGGGCTTCT<br>RP: GCAGTACAGTCTACAAGCCT |

6. Primers for examining Cre-mediated excisions in plants.

| Primer | Sequence (5’ to 3’) |
|---|---|
| 35S-F | ACGCACAATCCCACTATCCTTC |
| UBI-R | GGACCGGAACGCCGATCTAGAG |
| Cre-F | GTACAGTATAGGCTTTCCCG |
| PMI-R | CCCTGCATATTCAACAGGCTG |

7. Primers for T-DNA identification in progenies.

| Primer | Sequence (5’ to 3’) |
|---|---|
| OsMLH1-RNAi-F | GTACGGACCGTACTACTCTA |
| OsMLH1-RNAi-R | TTATCCTAGTTTGCGCGCTA |
| Cas9-F | CGTGGATGAGGTGGCCTACC |
| Cas9-R | CGAGGTCGTCGTCGTAGGTG |

**Supplemental Sequence.**

>Conditional excision RNAi module

ggtttgcgtattggctagagcagcttgccaacatggtggagcacgacactctcgtctactccaagaatatcaaagatac
agtctcagaagaccaaagggctattgagacttttcaacaaagggtaatatcgggaaacctcctcggattccattgccc
agctatctgtcacttcatcaaaggacagtagaaaaggaaggtggcacctacaaatgccatcattgcgataaagga
aaggctatcgttcaagatgcctctgccgacagtggtcccaaagatggacccccacccacgaggagcatcgtggaaa
aagaagacgttccaaccacgtcttcaaagcaagtggattgatgtgaacatggtggagcacgacactctcgtctactcc
aagaatatcaaagatacagtctcagaagaccaaagggctattgagacttttcaacaaagggtaatatcgggaaacct
cctcggattccattgcccagctatctgtcacttcatcaaaggacagtagaaaaggaaggtggcacctacaaatgcca
tcattgcgataaaggaaaggctatcgttcaagatgcctctgccgacagtggtcccaaagatggacccccacccacga
ggagcatcgtggaaaaagaagacgttccaaccacgtcttcaaagcaagtggattgatgtgatatctccactgacgtaa
gggatgacgcacaatcccactatccttcgcaagacccttcctctatataaggaagttcatttcatttggagaggacacgc
tgaaatcaccagtctctctctacaaatctatctctGGTACCataacttcgtataatgtatgctatacgaagttatgtcgac
attaccctgttatccctatgcagtgcagcgtgacccggtcgtgcccctctctagagataatgagcattgcatgtctaagtta
taaaaaattaccacatattttttgtcacacttgtttgaagtgcagtttatctatctttatacatatatttaaactttactctacga
ataatataatctatagtactacaataatatcagtgttttagagaatcatataaatgaacagttagacatggtctaaaggac
aattgagtattttgacaacaggactctacagttttatctttttagtgtgcatgtgttctcctttttttttgcaaatagcttcacctata
taatacttcatccattttattagtacatccatttagggtttagggttaatggtttttatagactaattttttagtacatctattttattct
attttagcctctaaattaagaaaactaaaactctattttagtttttttatttaataatttagatataaaatagaataaaataaagt
gactaaaaattaaacaaataccctttaagaaattaaaaaaactaaggaaacatttttcttgtttcgagtagataatgcca
gcctgttaaacgccgtcgacgagtctaacggacaccaaccagcgaaccagcagcgtcgcgtcgggccaagcgaa
gcagacggcacggcatctctgtcgctgcctctggacccctctcgagagttccgctccaccgttggacttgctccgctgtc
ggcatccagaaattgcgtggcggagcggcagacgtgagccggcacggcaggcggcctcctcctcctctcacggca
ccggcagctacgggggattcctttcccaccgctccttcgctttcccttcctcgcccgccgtaataaatagacaccccctcc
acaccctctttccccaacctcgtgttgttcggagcgcacacacacacaaccagatctcccccaaatccacccgtcggc
acctccgcttcaaggtacgccgctcgtcctcccccccccccctctctaccttctctagatcggcgttccggtccatggttag
ggcccggtagttctacttctgttcatgtttgtgttagatccgtgtttgtgttagatccgtgctgctagcgttcgtacacggatgc
gacctgtacgtcagacacgttctgattgctaacttgccagtgtttctctttggggaatcctgggatggctctagccgttccgc
agacgggatcgatttcatgattttttttgtttcgttgcatagggtttggtttgcccttttcctttatttcaatatatgccgtgcacttgt
ttgtcgggtcatcttttcatgcttttttttgtcttggttgtgatgatgtggtctggttgggcggtcgttctagatcggagtagaattct
gtttcaaactacctggtggatttattaattttggatctgtatgtgtgtgccatacatattcatagttacgaattgaagatgatgg
atggaaatatcgatctaggataggtatacatgttgatgcgggttttactgatgcatatacagagatgcttttttgttcgcttggt
tgtgatgatgtggtgtggttgggcggtcgttcattcgttctagatcggagtagaatactgtttcaaactacctggtgtatttatt
aattttggaactgtatgtgtgtgtcatacatcttcatagttacgagtttaagatggatggaaatatcgatctaggataggtat
acatgttgatgtgggttttactgatgcatatacatgatggcatatgcagcatctattcatatgctctaaccttgagtacctatct
attataataaacaagtatgttttataattattttgatcttgatatacttggatgatggcatatgcagcagctatatgtggattttttt
agccctgccttcatacgctatttatttgcttggtactgtttcttttgtcgatgctcaccctgttgtttggtgttacttctgcactaAgt
accTTGAGGATTTGGCAATATTGTGCGAAAGGCATACTACCTCAAAGTTATCTGCAT
ACGAGGATCTGCAGACCATAAAATCGATGGGGTTCAGAGGGGAGGCTTTGGCTA
GTATGACTTATGTTGGCCATGTTACCGTGACAACGATAACAGAAGGCCAATTGCA
CGGCTACAGGGTTTCTTACAGAGATGGTGTAATGGAGAATGAGCCTAAGCCTTG
CGCTGCGGTGAAAGGAACTCAAGTCATGGTTGAAAATCTATTTTACAACGTACGG
ACCGTACTACTCTATTCGTTTCAATATATTTATTTGTTTCAGCTGACTGCAAGATTC

AAAAATTTCTTTATTATTTTAAATTTTGTGTCACTCAAAACCAGATAAACAATTTGAT
ATAGAGGCACTATATATATACATATTCTCGATTATATATGTAAATGAGTTAACCTTTTT
TTCCACTTAAATTATATAGGTTGTAAAATAGATTTTCAACCATGACTTGAGTTCCTTT
CACCGCAGCGCAAGGCTTAGGCTCATTCTCCATTACACCATCTCTGTAAGAAACC
CTGTAGCCGTGCAATTGGCCTTCTGTTATCGTTGTCACGGTAACATGGCCAACAT
AAGTCATACTAGCCAAAGCCTCCCCTCTGAACCCCATCGATTTTATGGTCTGCAG
ATCCTCGTATGCAGATAACTTTGAGGTAGTATGCCTTTCGCACAATATTGCCAAAT
CCTCAAggatccccgggaattctaagaggagtccaccatggtagatctgactagtgttaacgctagccaccacca
ccaccaccacgtgtgaattacaggtgaccagctcgaatttccccgatcgttcaaacatttggcaataaagtttcttaagat
tgaatcctgttgccggtcttgcgatgattatcatataatttctgttgaattacgttaagcatgtaataattaacatgtaatgcat
gacgttatttatgagatgggtttttatgattagagtcccgcaattatacatttaatacgcgatagaaaacaaaatatagcgc
gcaaactaggataaattatcgcgcgcggtgtcatctatgttactagatcagcttacagaaggagcagctagaacggtc
atatatgcatgcatatgcaatgcgtgcatgctaagggtcagtgtattatttttatagttttgaatcaatactacaatatttaacg
atactgtgatattgatggcaacaagatgtttggccggtatatgtaaaatatagtttcagatactatgatttattcaaaactat
actatttttagagttgtagaaactacacttagaaccttagtttttttcttttctcttacatatattttggtttttaatggagccaaac
aaatcttaattctaagcaatactttgattttactgtgataagatagtactgtaatatttatattatatataattataaactataata
tttcaaaactatagtattttaaaattgcattaacaaacatgtcctaattggtactcctgagatactataccctcctgttttaaaa
tagttggcattatcgaattatcattttactttttaatgttttctcttcttttaatatattttatgaattttaatgtattttaaaatgttatgca
gttcgctctggacttttctgctgcgcctacacttgggtgtactgggcctaaattcagcctgaccgaccgcctgcattgaata
atggatgagcaccggtaaaatccgcgtacccaactttcgagaagaaccgagacgtggcgggccgggccaccgac
gcacggcaccagcgactgcacacgtcccgccggcgtacgtgtacgtgctgttccctcactggccgcccaatccactc
atgcatgcccacgtacacccctgccgtggcgcgcccagatcctaatcctttcgccgttctgcacttctgctgcctataaat
ggcggcatcgaccgtcacctgcttcaccaccggcgagccacatcgagaacacgatcgagcacacaagcacgaag
actcgtttaggagaaaccacaaaccaccaagccgtgcaagcaccatggtgcccaagaagaagaggaaagtctcg
aatctcctgactgttcaccagaatctccccgccctgcccgttgatgctacaagcgatgaggtccgcaagaatctgatgg
atatgttcagggaccggcaggccttctctgagcacacctggaagatgctcctgtcagtgtgcaggtcctgggctgcttgg
tgcaagctgaacaataggaagtggttcccagcggagcctgaggacgttcgcgattacctcctgtacctgcaggctag
gggcctcgctgtcaagacgattcagcagcacctcggccagctgaacatgctccataggaggtccgggctgccaagg
ccatccgacagcaatgccgtgagcctcgtcatgaggaggatcaggaaggagaacgtggatgctggcgagagggct
aagcaggctctggctttcgagaggaccgacttcgaccaggtaagtcttcttttcctttactctttacagaaatggtaatctc
agatatagtaatggataagatccaaaaatgacacttttaaccaagattgtacgaagatctttttaaactccatttttttatttttg
acatctaaattggatttaactcggccttgctgtattttggcaggttcgtagcctcatggagaactcggaccgctgccagga
tattaggaatctggcgttcctcggcatcgcttacaacacactcctgcgcatcgcggagattgctcgcatcagggtcaag
gacatctcccggactgatggtggccgcatgctgatccatattggccgcaccaagacgctcgtttctaccgctggcgtgg
agaaggctctctcactgggggtgacgaagctggtcgagaggtggatctcggtttctggcgtggccgacgatccgaac
aattacctcttctgccgggtccgcaagaatggggttgctgccccctcagcgacatcccagctgagcactagggctctcg
agggcattttcgaggctacacacaggctgatctacggcgccaaggacgattcggggcagagAtacctcgcttggtcg
ggccattctgctagggtgggggctgctagggacatggctagggctggcgtctccattccggagatcatgcaggctggc
ggctggaccaacgtcaatatcgttatgaactacattcggaacctggactcggagactggggcgatggtgcggctgctg
gaggacggcgattgaaactgagtcgagctcaggcctcccagctttcgtccgtatcatcggtttcgacaacgttcgtcaa
gttcaatgcatcagtttcattgcccacacaccagaatcctactaagtttgagtattatggcattggaaaagctgttttcttcta
tcatttgttctgcttgtaatttactgtgttctttcagtttttgttttcggacatcaaaatgcaaatggatggataagagttaataaa
tgatatggtccttttgttcattctcaaattattattatctgttgtttttactttaatgggttgaatttaagtaagaaaggaactaaca
gtgtgatattaaggtgcaatgttagacatataaaacagtctttcacctctctttggttatgtcttgaattggtttgtttcttcactta

tctgtgtaatcaagtttactatgagtctatgatcaagtaattatgcaatcaagttaagtacagtataggctttCCCGGGa
gggataacagggtaatgtcgacataacttcgtataatgtatgctatacgaagttatGGTACCatgcaaaaactcatt
aactcagtgcaaaactatgcctggggcagcaaaacggcgttgactgaactttatggtatggaaaatccgtccagcca
gccgatggccgagctgtggatgggcgcacatccgaaaagcagttcacgagtgcagaatgccgccggagatatcgtt
tcactgcgtgatgtgattgagagtgataaatcgactctgctcggagaggccgttgccaaacgctttggcgaactgccttt
cctgttcaaagtattatgcgcagcacagccactctccattcaggttcatccaaacaaacacaattctgaaatcggttttgc
caaagaaaatgccgcaggtatcccgatggatgccgccgagcgtaactataaagatcctaaccacaagccggagct
ggtttttgcgctgacgcctttccttgcgatgaacgcgtttcgtgaattttccgagattgtctccctactccagccggtcgcag
gtgcacatccggcgattgctcactttttacaacagcctgatgccgaacgtttaagcgaactgttcgccagcctgttgaata
tgcagggtgaagaaaaatcccgcgcgctggcgattttaaaatcggccctcgatagccagcagggtgaaccgtggca
aacgattcgtttaatttctgaattttacccggaagacagcggtctgttctccccgctattgctgaatgtggtgaaattgaacc
ctggcgaagcgatgttcctgttcgctgaaacaccgcacgcttacctgcaaggcgtggcgctggaagtgatggcaaact
ccgataacgtgctgcgtgcgggtctgacgcctaaatacattgatattccggaactggttgccaatgtgaaattcgaagc
caaaccggctaaccagttgttgacccagccggtgaaacaaggtgcagaactggacttcccgattccagtggatgattt
tgccttctcgctgcatgaccttagtgataaagaaaccaccattagccagcagagtgccgccattttgttctgcgtcgaag
gcgatgcaacgttgtggaaaggttctcagcagttacagcttaaaccgggtgaatcagcgtttattgccgccaacgaatc
accggtgactgtcaaaggccacggccgtttagcgcgtgtttacaacaagctgtaactcgagtttctccataataatgtgt
gagtagttcccagataagggaattagggttcctatagggtttcgctcatgtgttgagcatataagaaacccttagtatgtat
ttgtatttgtaaaatacttctatcaataaaatttctaattcctaaaaccaaaatccagtactaaaatccagatc

The sequences were labeled as follow:

CaMV 35S-LoxP-ZmUBIp-sense *OsMLH1*-intron spacer-antisense *OsMLH1*-NOS Ter-Rab17p-*intronized Cre*-3AT Ter-LoxP-*PMI*-35S Ter
