# Supplementary material for: Conditional knockdown of OsMLH1 to improve plant prime editing systems without disturbing fertility in rice
Source: Genome Biol. 2024 May 21;25:131. doi: 10.1186/s13059-024-03282-y (PMC11110357; doi:10.1186/s13059-024-03282-y)
Supplement: Supplementary file 2 — Additional file 2. Review history. [file 13059_2024_3282_MOESM2_ESM.docx]

Review History

**First round of review**

**Reviewer 1**

**Are you able to assess all statistics in the manuscript, including the appropriateness of statistical tests used?** No, I do not feel adequately qualified to assess the statistics.

**Comments to author:**

Authors showed that knockdown of OsMLH1 by RNAi greatly and stably outperforms inhibition of OsMLH1 activity via co-expressing a dominant negative variant of OsMLH1 to enhance prime editing in rice plants. This is exciting news for improving efficiency of prime editing by inhibition of MMR. To overcome partial sterility resulting from OsMLH1 knockdown, authors developed a conditional excision system to remove the RNAi module by Cre mediated site-specific recombination. Authors demonstrated a feasible and comprehensive strategy to solve the problems encountered when enhancing prime editing via inhibition of MMR.

Minor revisions:

1. Compared to ePE3 and ePE5c-Cre, seed-setting rate of ePE5c lines was much lower. However, in theory, many seeds of ePE5c lines should be T-DNA free. Can you provide the data for ratio of T-DNA-free seeds to total number of seeds from ePE5c?

2. Gupta et al. (2023) reported that whole-genome sequencing detected no OsMLH1dn-associated random mutations, please discuss their results. (Gupta, A. et al. Plant Biotechnol. J. 2023, 21:1454-1464).

3. Please provide the data for fertility of ePE5a/b if available.

4. Are the prime editing targets in this report agriculturally important? If so, please discuss the traits of interest for the edited plants.

5. Expression cassette sequences of RNAi and the conditional excision system should be presented in Supplemental Materials.

6. Language and writing should be checked thoroughly, e.g.:

Abstract: "The study provide" and "To overcome partially sterile"

P12L3: ePE5c? ePE5c-Cre?

P15L15: it? Its?

**Reviewer 2**

**Are you able to assess all statistics in the manuscript, including the appropriateness of statistical tests used?** Yes, and I have assessed the statistics in my report.

**Comments to author:**

As an important precise gene editing tool, the high efficiency of the prime editing (PE) system is crucial for its application in crop breeding. In this manuscript, Liu et al. reported a safe and reliable plant PE optimization strategy to improve the editing efficiency without disturbing plant development. The results show that the editing efficiency of all sites has been significantly improved, with editing efficiency reaching 100% at some sites. This system provides a significant help for the better application of PE system to agricultural practice. I have some minor comments:

1. In Figure 1a, reverse transcriptase should not be represented by MMLV, but by the NC protein and the RNase H domain.

2. In Figure 1b, the description of editing efficiency assessment in callus tissue is unclear. It is unclear how the three biological replicates were conducted.

3. In Figure 2cde and 4ef, detailed descriptions of the methodology and corresponding plant numbers should be provided for assessing the fertility phenotype and data statistics.

4. There is an error in the legend of Figure 2b. Homozygous should be represented by red, and heterozygous by blue.

5. In Figure 2b, the definition of "non-homozygous" is not clear. Does it refer to heterozygous?

6. In Figure 1a, it is recommended to provide detailed vector construction information for the RNAi construct of OsMLH1 to facilitate reproducibility of experiments by readers.

7. Arabidopsis in Page 3 line 16, hMLH1dn in Page 5 line 6 and Agrobacterium in Page 5 line 21 should be italicized.

**Authors Response**

**Point-by-point responses to the reviewers’ comments:**

Reviewer #1:

Remarks: Authors showed that knockdown of OsMLH1 by RNAi greatly and stably outperforms inhibition of OsMLH1 activity via co-expressing a dominant negative variant of OsMLH1 to enhance prime editing in rice plants. This is exciting news for improving efficiency of prime editing by inhibition of MMR. To overcome partial sterility resulting from OsMLH1 knockdown, authors developed a conditional excision system to remove the RNAi module by Cre mediated site-specific recombination. Authors demonstrated a feasible and comprehensive strategy to solve the problems encountered when enhancing prime editing via inhibition of MMR.

*Response: We highly appreciate the constructive suggestions and valuable comment from Reviewer #1. The revisions have been made as follows.*

Minor revisions:

Comment 1. Compared to ePE3 and ePE5c-Cre, seed-setting rate of ePE5c lines was much lower. However, in theory, many seeds of ePE5c lines should be T-DNA free. Can you provide the data for ratio of T-DNA-free seeds to total number of seeds from ePE5c?

*Response: Thank you for the suggestion. We are interested in the possibility of OsMLH1-related T-DNA segregation preference. It has been reported that OsMLH1 is involved in meiosis regulation, but no evidence has shown that it also regulates the development of gametes. According to this suggestion, we assessed the T1 progenies of four T0 ePE5c lines. As shown in Supplemental Table S4, enrichment of T-DNA-free seeds was not observed. Because a reduction in the seed-setting rate results from defective crossover in mlh1 mutants, it is not surprising that the RNAi impaired the overall generation of the embryo sac and/or pollen and did not alter the segregation of T-DNA.*

Comment 2. Gupta et al. (2023) reported that whole-genome sequencing detected no OsMLH1dn-associated random mutations, please discuss their results. (Gupta, A. et al. Plant Biotechnol. J. 2023, 21:1454-1464).

*Response: We apologize for missing key literature. Following this suggestion, the reference has been added to the main text.*

Comment 3. Please provide the data for fertility of ePE5a/b if available.

*Response: Thank you for the comment. Given that ePE5a/b did not robustly outperform ePE3 at most targets, stable transgenic plants of ePE5a/b for the six targets were not obtained by retransformation in this study. In addition, we generated some ePE5a/b transgenic plants with normal fertility at targets besides of this report. Because the pegRNAs and rice varieties used for ePE5a/b editing differ from those used for ePE3 and ePE5c editing, these data may be less appropriate due to the lack of strict controls. However, we would like to provide or to discuss the related data if necessary.*

Comment 4. Are the prime editing targets in this report agriculturally important? If so, please discuss the traits of interest for the edited plants.

*Response: Thank you for the comment. Although some of the targeted genes are agriculturally important, we did not use FNP sites in this proof-of-concept study for tool optimization.*

Comment 5. Expression cassette sequences of RNAi and the conditional excision system should be presented in Supplemental Materials.

*Response: Thank you for the comment. The expression cassette sequences used for RNAi and the conditional excision system have been added as supplemental sequences in the revised manuscript.*

Comment 6. Language and writing should be checked thoroughly, e.g.:

Abstract: "The study provide" and "To overcome partially sterile"

P12L3: ePE5c? ePE5c-Cre?

P15L15: it? Its?

*Response: We apologize for the language mistakes. To improve the writing quality, we carefully read through the manuscript to avoid any confusing expressions or writing issues. In addition, the professional English service “American Journal Experts” helped us polish the language.*

Reviewer #2:

Remarks: As an important precise gene editing tool, the high efficiency of the prime editing (PE) system is crucial for its application in crop breeding. In this manuscript, Liu et al. reported a safe and reliable plant PE optimization strategy to improve the editing efficiency without disturbing plant development. The results show that the editing efficiency of all sites has been significantly improved, with editing efficiency reaching 100% at some sites. This system provides a significant help for the better application of PE system to agricultural practice.

*Response: We highly appreciate the encouragement and kind comments from Reviewer #2. The revisions have been made as follows.*

Comment 1. In Figure 1a, reverse transcriptase should not be represented by MMLV, but by the NC protein and the RNase H domain.

*Response: We apologize for the misleading labeling. Figure 1a has been revised accordingly.*

Comment 2. In Figure 1b, the description of editing efficiency assessment in callus tissue is unclear. It is unclear how the three biological replicates were conducted.

*Response: We appreciate this comment. As shown in Figure 1b, stably transformed calli were used for efficiency assessments. After two to three weeks of selection, approximately 200 newly emerged calli were collected for DNA extraction. Independent transformations with different Agrobacterium clones were performed as biological replicates. Amplicon NGS was performed with site-specific primers to produce more than 0.5 Gb of data per sample. The efficiencies were calculated from the ratio of edited reads to total clean reads. The related descriptions have been added to the figure legends and the Materials and Methods section to improve the readability of the manuscript.*

Comment 3. In Figure 2cde and 4ef, detailed descriptions of the methodology and corresponding plant numbers should be provided for assessing the fertility phenotype and data statistics.

*Response: We appreciate this comment. In the legends of Figures 2 and 4, the methodology details and plant numbers were showed for the statistical analysis in the revised manuscript. We can also provide the seed-setting ratio of every single line if necessary.*

Comment 4. There is an error in the legend of Figure 2b. Homozygous should be represented by red, and heterozygous by blue.

*Response: We sincerely apologize for this mistake. This issue has been corrected in the revised version.*

Comment 5. In Figure 2b, the definition of "non-homozygous" is not clear. Does it refer to heterozygous?

*Response: We apologize for the confusion. The non-homozygous plants contained heterozygous lines with precise edits, and the lines simultaneously carried precise edits and byproduct(s). A further explanation has been added to the figure legend. We hope these revisions have clarified any confusion.*

Comment 6. In Figure 1a, it is recommended to provide detailed vector construction information for the RNAi construct of OsMLH1 to facilitate reproducibility of experiments by readers.

*Response: We appreciate this comment. The detailed sequences of the RNAi cassette and the conditional excision system have been provided in the Supplemental Materials. In addition, we are uploading the ePE5c and ePE5c-cre binary vectors to Addgene. We hope that the plasmids and construction information could help the improvement of plant PE systems.*

Comment 7. Arabidopsis in Page 3 line 16, hMLH1dn in Page 5 line 6 and Agrobacterium in Page 5 line 21 should be italicized.

*Response: We apologize for the errors. They have been revised as suggested. The manuscript has been carefully checked to avoid formatting and writing issues.*

**Second round of review**

**Reviewer 1**

All my concerns were addressed. No further comments.
